# DExplore: An Online Tool for Detecting Differentially Expressed Genes from mRNA Microarray Experiments

**DOI:** 10.3390/biology13050351

**Published:** 2024-05-16

**Authors:** Anna D. Katsiki, Pantelis E. Karatzas, Hector-Xavier De Lastic, Alexandros G. Georgakilas, Ourania Tsitsilonis, Constantinos E. Vorgias

**Affiliations:** 1 Department of Biology, School of Science, National and Kapodistrian University of Athens, 15784 Athens, Greece; 2 Unit of Process Control and Informatics, Department of Process Analysis and Plant Design, School of Chemical Engineering, National Technical University of Athens (NTUA), Zografou Campus, 15780 Athens, Greece; 3 DNA Damage Laboratory, Physics Department, School of Applied Mathematical and Physical Sciences, National Technical University of Athens (NTUA), Zografou Campus, 15780 Athens, Greece

**Keywords:** web application, bioinformatics, differential expression analysis, gene expression, gene enrichment analysis, microarrays, response to chemotherapeutics, gene expression profile

## Abstract

**Simple Summary:**

DExplore is a user-friendly web application addressing challenges in analyzing mRNA microarray experiments for gene expression profiling. For over fifteen years, microarray experiments have played a vital role in understanding gene expression in various conditions. DExplore, built with the R programming language, Shiny package, and Bioconductor, caters to researchers lacking programming skills. It facilitates the detection of differentially expressed genes using data from the NCBI Gene Expression Omnibus (GEO) and integrates with WebGestalt for functional enrichment analysis. DExplore incorporates visualization plots, enhancing the interpretability of analysis results. These plots provide researchers with deeper insights into gene expression patterns, facilitating informed decision-making. The application’s versatility extends to a Docker image available on Docker Hub, enabling local usage of data not submitted to GEO. Currently tailored for the Affymetrix platforms, DExplore serves as a powerful application, simplifying the analysis of high-throughput data. This online accessible tool empowers molecular biologists to focus on a smaller number of biologically relevant genes, eliminating complexity and enhancing the utility of publicly available data in advancing gene expression research. DExplore is a free and open-source web application.

**Abstract:**

Microarray experiments, a mainstay in gene expression analysis for nearly two decades, pose challenges due to their complexity. To address this, we introduce DExplore, a user-friendly web application enabling researchers to detect differentially expressed genes using data from NCBI’s GEO. Developed with R, Shiny, and Bioconductor, DExplore integrates WebGestalt for functional enrichment analysis. It also provides visualization plots for enhanced result interpretation. With a Docker image for local execution, DExplore accommodates unpublished data. To illustrate its utility, we showcase two case studies on cancer cells treated with chemotherapeutic drugs. DExplore streamlines microarray data analysis, empowering molecular biologists to focus on genes of biological significance.

## 1. Introduction

DNA microarrays are used to detect the presence and abundance of mRNA molecules in various biological samples [1]. Thus, they can be used in the following ways: (a) to measure gene expression levels on a whole-genome scale at a specific time-point; (b) to compare gene expression in different conditions; (c) to study responses to drugs or other treatments, such as genotoxic agents like chemicals or radiation; and (d) to obtain useful information on the biological function of an organism or tissue by identifying the genes that are activated or suppressed at various developmental stages or in response to environmental stimuli [2].

Despite the development of new technologies, such as RNA sequencing, microarray technology remains one of the most powerful and affordable methods for identifying and analyzing cellular signaling pathways. The ability to simultaneously analyze thousands of transcripts provides a detailed molecular phenotype, which can be used to deduce the different subsets of responses affected upon activation or inhibition of a signaling pathway. Moreover, in recent years, datasets from microarray experiments are increasingly utilized for re-analyses (e.g., [3,4]) or meta-analyses (e.g., [5,6]) in the context of systems biology approaches. Thus, the analysis of mRNA microarray experiments for detecting gene expression profiles remains a common process for biologists and biomedical researchers.

A microarray experiment produces one set of images that are transformed into numerical values, representing absolute (single-channel) or relative (two-channel) intensities depending on the array used. The quality of raw data needs to be examined and assessed; further data preprocessing is required to reach the gene expression matrix. Essentially, there are two or three steps depending on the type of array: a background adjustment to remove signals due to non-specific hybridization of the probe; normalization to correct systematic (non-biological) signals, such as different dye absorption, and spatial heterogeneity on the chip. The normalization is usually performed assuming that only a relatively small number of genes is differentially expressed and that these genes are equally under- or over-expressed. In the Affymetrix (Santa Clara, CA, USA) arrays, it is also necessary to summarize the different signals obtained from all the probes representing one gene in a unique value. The aforementioned steps generate the gene expression matrix, wherein rows represent the genes and columns represent the samples. Each data value (a cell in the gene expression matrix) represents the expression level of a gene (row) in a sample (column). The numeric value per se is not that important. However, the relative expression level of a gene in various samples is valuable in this analysis. Subsequently, the gene expression matrices are used to detect the genes that are differentially expressed among treated samples.

Currently, a growing number of computational tools for analyzing the output of microarray experiments is available. Undoubtedly, the most widely used among them are R programming language [7] and Bioconductor [8]. Both are open source and provide the ability of multiple modifications depending on the user’s requirements. The main disadvantages of R and Bioconductor are that users need to be familiar with programming and that the tools required for the analysis need to be downloaded and installed on their computer. For these reasons, a number of tools, commercial or freely available, have been developed for wet lab biologists and researchers.

Since microarray analyses have been conducted for more than 15 years, many online tools, such as GEPAS [9], EzArray [10], Expression Profiler [11], MIDAW [12], ArrayNorm [13], and ArrayPipe [14], are no longer updated or may not even be available. On the other hand, Babelomics [15] and CARMAweb [16] are some examples of widely used online applications that are freely available to non-commercial users. These applications require researchers to upload raw data stored in their computer and adjust parameters for analysis. The process of uploading raw data is typically time-consuming, even for experiments with a small number of replicates, and utilizing these tools can be intricate. Currently, GEO2R [17] emerges as the most widely used tool for analyzing microarray data. GEO2R is integrated into the NCBI Gene Expression Omnibus (GEO) database and can be easily used online to identify differentially expressed genes (DEGs) between two experimental conditions.

In this paper, we introduce DExplore, an online user-friendly web application designed for the detection of DEGs using data from NCBI GEO [17,18]. GEO is an international public repository that archives and freely distributes high-throughput functional genomics data submitted by the research community. DExplore is also equipped for local data analysis, even for datasets not submitted to NCBI’s GEO, because of a Docker image built for the application. This feature ensures privacy for users who have not yet submitted their data and seek a preliminary estimation of their experimental results. The capability for local analysis is particularly advantageous in cases where the server’s upload limitations may affect the ease and speed of data processing.

Moreover, our application facilitates a seamless transition to functional enrichment analysis using the well-established online tool, WebGestalt [18]. Users can utilize the results obtained from DExplore’s preceding differential expression analysis directly in WebGestalt. Additionally, the tool provides graphical representations since visualization plots help users interpret results better than large lists of genes and statistical values. DExplore generates histograms, boxplots, interactive heatmaps, interactive volcano plots, and PCA plots (including scree plots, PCA scores plots, and biplots) in a .zip file for users to download and explore. 

To showcase DExplore’s versatility, we present two examples involving human breast cancer cells treated with doxorubicin (GEO Series: GSE39870 [19]—https://www.ncbi.nlm.nih.gov/geo/query/acc.cgi?acc=GSE39870, accessed on 17 April 2024; and GSE113427 [20,21]—https://www.ncbi.nlm.nih.gov/geo/query/acc.cgi?acc=GSE113427, accessed on 17 April 2024). The results of the analyses with DExplore, followed by the functional enrichment analysis with WebGestalt, are compared to those obtained from GEO2R. We also conducted a comparison with CARMAweb. Additionally, the findings are shown to be consistent with cellular pathways reported in the literature for this specific chemotherapeutic agent. At present, DExplore is capable of analyzing single-channel mRNA microarray experiments performed by the Affymetrix platforms for all organisms. In the near future, it will be extended to include other commercially available platforms, such as Illumina and Agilent.

In conclusion, DExplore stands as a robust application, serving as a valuable reference. It simplifies the complexities of publicly available high-throughput data, allowing molecular biologists to focus on the selection of few genes with biological relevance for their experiments without requiring any programming skills. 

DExplore is available at www.dexplore.gr, accessed on 19 April 2024, and the source code can be found at https://github.com/annakatsiki/dexplore, accessed on 19 April 2024. 

## 2. Materials and Methods

DExplore can be used to perform all steps of a classical differential expression analysis (DEA), including data retrieval from GEO, data preprocessing, and differential expression analysis through an empirical Bayes-moderated *t*-test. The results provide a list of the DEGs annotated with metadata as well as graphical representations for visual inspection of the data and the analysis results. Additionally, users can directly utilize functional enrichment analysis with WebGestalt to identify enriched gene ontology (GO) terms [22,23]. 

### 2.1. Implementation

DExplore is a web application built using the R programming language, Shiny, and Bioconductor, making it accessible to researchers without requiring advanced programming skills. The primary R and Bioconductor packages employed in implementing DExplore include the following: (a) Shiny [24]—for constructing interactive web applications; (b) GEOquery [25]—for downloading raw data from the NCBI GEO; (c) oligo [26]—for the analysis of gene expression raw-level data (microarray preprocessing); (d) limma [27]—for data analysis, linear modeling, and differential expression analysis; (e) annotate [28]—for annotating the identified DEGs; (f) the annotation package corresponding to each Affymetrix platform to be analyzed, as provided by Bioconductor. In the development of the application, we also utilized the shinyjs [29], shinyBS [30], and DT [31] packages. For the visualization plots, we used ggplot2 [32] for the static image plots and heatmaply [33] and plotly [34] for the interactive heatmaps and volcano plots, respectively. 

Furthermore, as many researchers continue to rely on microarray experiments for exploring and comparing molecular pathways activated or inactivated after a specific treatment, we integrated the WebGestaltR package [35] to enable direct functional enrichment analysis. Within the tab panel labeled “WebGestalt Over-Representation Analysis (ORA)”, users can conveniently proceed to the WebGestalt ORA using the list of DEGs identified by DExplore. The functional database employed for the analysis is GO [22,23], encompassing all three categories: Biological Process, Molecular Function, and Cellular Component.

Over the last ten years, the necessity to develop and distribute software tools among developers and users, as well as among developers themselves, has driven the development of Docker and Kubernetes. Containerization using Docker allows the packaging, shipping, and running of entire applications along with their dependencies on any computer [36,37,38]. Recognizing the potential need for researchers to use our tool locally, we created a Docker image of DExplore, which is available for direct download from Docker Hub. This enables users to employ the application on their computer’s operating system without the need to install the R programming language, R, or Bioconductor packages, and without requiring any knowledge of R programming.

### 2.2. Tool Overview

DExplore offers users versatility, enabling seamless analysis whether conducted online or locally. Its user interface is designed to be straightforward and user-friendly. This tool is freely accessible to all users, requiring only an internet connection and a browser and eliminating the need for installation or registration. Users have the flexibility to utilize data from the NCBI GEO database or upload raw data generated from the Affymetrix GeneChips in .CEL file format. When using the platform online with uploaded .CEL files, it is important to note that data is temporarily stored in the server and automatically deleted upon a user’s exit or session expiration, ensuring data security. Due to file size limitations for uploaded .CEL files, we strongly recommend that users employ DExplore with uploaded data and run the Docker image on their infrastructure following the instructions on the image’s webpage. Running DExplore through the locally installed Docker image prevents any risk of data leakage. 

DExplore is organized into four main tab panels, Data Input, Data Description, Results, and WebGestalt Over-Representation Analysis, along with a user’s guide and contact email in the “About” tab. The user’s guide for DExplore is provided as Supplementary File S1.

**Figure 1:**
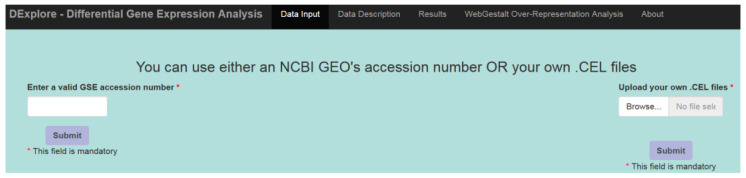
DExplore’s layout, featuring four main tab panels: Data Input, Data Description, Results, and WebGestalt Over-Representation Analysis. The “About” tab provides Supplementary Information, including a user’s guide and contact details.

As input, users can provide a GSE accession number from the NCBI GEO database or upload data (.CEL files) from their computer (Figure 1). For GSE input, DExplore validates the query accession number and provides a hyperlink to the corresponding GEO page.

**Figure 2:**
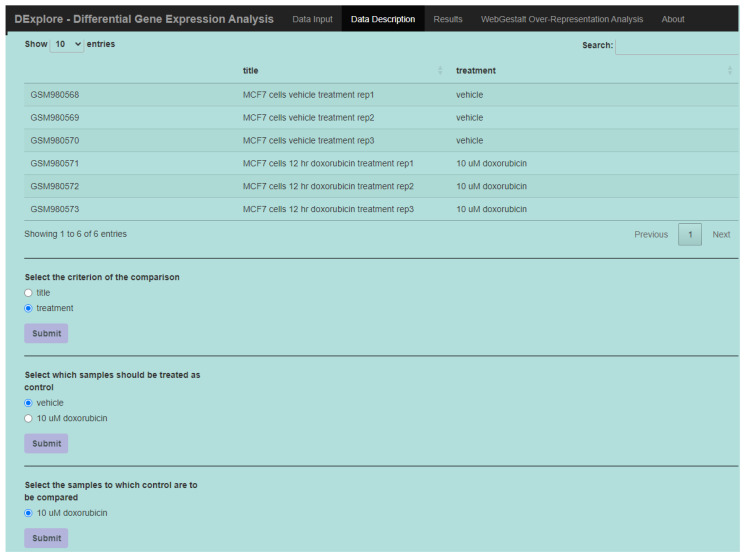
The “Data Description” tab. Experimental design information is retrieved directly from GEO when a GSE input is provided (in this case, GSE39870). Users have the option to select the comparison criterion, such as type of treatment, and designate which samples should serve as control and which are treated as samples.

DExplore enables the user to customize the analysis by choosing the comparison to be performed. The “Data Description” tab enables users to provide experimental design details, specify the platform for analysis (if multiple platforms exist in the same GSE), and define controls and treatment specifics (e.g., type of treatment, duration and concentration of chemical substances, dose of radiation) (Figure 2). 

In the context of gene expression analysis, we often compare the expression levels of thousands of genes simultaneously. When performing individual statistical tests for each gene without corrections, the probability of making at least one Type I error (false positive) becomes unacceptably high. As a result, running adjustments for multiple comparisons is essential when analyzing data from microarray experiments to control the overall Type I error rate in order to maintain the overall significance level. There are various methods for adjusting *p*-values to control the proportion of false discoveries among the significant results [39]. 

DExplore allows users to select an adjustment method for their analysis among the Bonferroni correction (“bonferroni”) [40], the correction introduced by Holm (1979) [41] (“holm”), by Hochberg (1988) [42] (“hochberg”), by Hommel (1988) [43] (“hommel”), by Benjamini & Hochberg (1995) [44] (“BH” or its alias “fdr”), and by Benjamini & Yekutieli 216 (2001) [45] (“BY”). A pass-through option (“none”) is also included.

The first four methods are designed to give strong control of the family-wise error rate (FWER). There seems to be no reason to use the unmodified Bonferroni correction, since it is overridden by Holm’s method, which is also valid under arbitrary assumptions. 

Hochberg’s and Hommel’s methods are valid when the hypothesis tests are independent or when they are non-negatively associated [46,47]. Hommel’s method is more powerful than Hochberg’s, but the difference is usually small, and the Hochberg *p*-values are faster to compute. 

The “BH” (also known as “fdr”) and “BY” method by Benjamini, Hochberg, and Yekutieli control the false discovery rate (FDR), which is the expected proportion of false discoveries amongst the rejected hypotheses. The false discovery rate is a less stringent condition than the family-wise error rate, and thus these methods are more powerful than the rest.

J.P. Shaffer has written a detailed review of commonly used adjustment methods [48]. 

**Figure 3:**
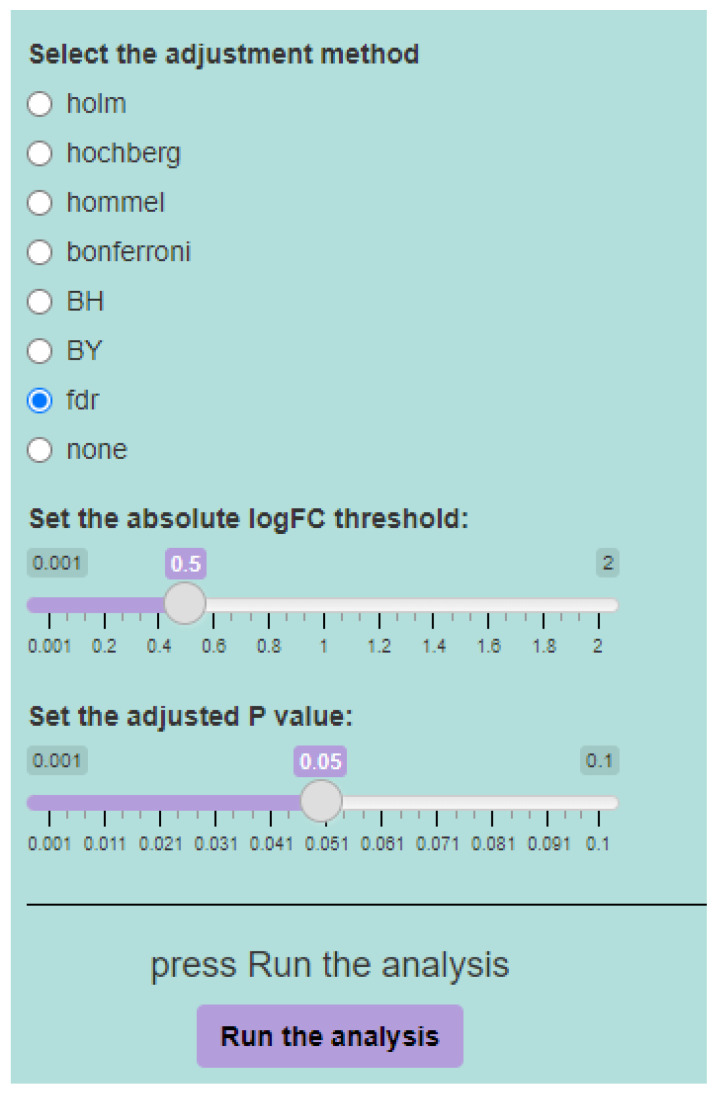
The statistical parameters for the differential expression analysis include the method for adjusting the *p*-value for multiple comparisons, the absolute log_2_ fold-change (FC) threshold, and the adjusted *p*-value threshold. Users can opt to use the default parameters, which employ false discovery rate (FDR) or “fdr” for multiple comparisons’ adjustment, 0.5 as the absolute log_2_ fold-change threshold, and 0.05 as the adjusted *p*-value threshold for the analysis. Alternatively, users have the flexibility to adjust these values based on their preferences.

Users can further tailor the analysis by selecting statistical parameters, including the method for adjusting multiple comparisons, absolute log_2_ fold-change threshold, and adjusted *p*-value threshold, ensuring the desirable statistical power (Figure 3). 

After selecting the parameters, the tool generates a list of DEGs. Users can then print, copy to clipboard, or download the list as a .csv or .tsv file for further analysis. Additionally, the tool provides visualization plots that can be downloaded as a .zip file. These include a histogram presenting the distribution of adjusted *p*-values plotted against the number of probes, a boxplot, an interactive heatmap, and an interactive volcano plot. Furthermore, DExplore generates three plots based on the results of a principal component analysis (PCA): a PCA plot depicting different sample groups based on PC1 and PC2, a scree plot, and a biplot.

### 2.3. DExplore’s Workflow

DExplore employs the GEOquery Bioconductor package [25] to download raw data and all necessary Supplementary Files for the analysis from GEO, or it uses the uploaded .CEL files. To read the .CEL files, the oligo package is utilized [26]. Raw data preprocessing is conducted using the RMA (Robust Multi-array Average) algorithm of the oligo package, employing default parameters such as background subtraction, quantile normalization, and summarization via median-polish [49,50,51].

Before advancing to linear modeling, DExplore conducts non-specific filtering using the pOverA method, where *p* represents the proportion of treated samples to the total number of samples, and A is log_2_100, an empirical value commonly used in such experiments. The linear modeling of the non-specific filtered values is performed using the limma package [27]. The user constructs the design matrix of the microarray experiment, determining which samples are treated as controls and which are target samples. To proceed with the analysis, there should be at least two replicates for each group. The lmFit function of the limma package fits a linear model to the expression data for each probe. The eBayes function computes moderated t-statistics, moderated F-statistic, and log-odds of the differential expression through the empirical Bayes moderation of the standard errors towards a global value [52]. The topTable function returns the genes that exceed the user-defined thresholds, i.e., absolute log_2_ fold-change and adjusted *p*-value thresholds computed by the chosen multiple correction method. 

In DExplore, the principal component analysis (PCA) is integrated using the prcomp function from the stats R package. The purpose of performing the PCA is to simplify high-dimensional microarray data by transforming them into a lower-dimensional space while retaining most of its variability. By identifying data patterns and relationships, the PCA aids in visualizing complex datasets and uncovering underlying structures. The PCA within DExplore generates PCA plots illustrating sample groupings based on PC1 and PC2 along with scree plots and biplots, providing enhanced data interpretations and insights. Furthermore, DExplore provides PCA plots generated with the ggplot2 R package along with other graphical representations, such as interactive heatmaps (heatmaply package), interactive volcano plots (plotly R package), histograms, and boxplots, to further facilitate data visualization and interpretation.

If the experiment’s platform has an annotation file in Bioconductor, the annotation is performed using the annotate package [28]. Unannotated DEGs are filtered out. In case no annotation file exists, DExplore provides a list of differentially expressed genes with their probe IDs instead of gene symbols. 

The optional step, functional enrichment analysis using WebGestalt’s overrepresentation analysis (ORA), is implemented through the WebgestaltR package [18], using the same parameters as WebGestalt’s web version.

While implementing functional enrichment analysis through overrepresentation analysis, it is crucial to be mindful of the significant impact that different gene reference background settings can have on enrichment *p*-values, even with the same statistical method and annotation content [53]. Therefore, configuring the gene reference background requires careful consideration. Although there is no universally accepted “gold” standard, a general guideline is to define it as the pool of genes selectable for the studied annotation category [54]. Additionally, choosing the right gene identifiers is vital for efficient mapping to available annotations. Ensuring comprehensive mapping for ID-to-ID and ID-to-annotation content in the database maximizes the translation of gene lists into potential annotation content for subsequent high-throughput enrichment analysis algorithms [54]. To avoid potential confusion from unannotated or inaccurately annotated probes, it is highly recommended to use probe IDs specific to the microarray platform used in the analyzed experiment.

### 2.4. Output

**Figure 4:**
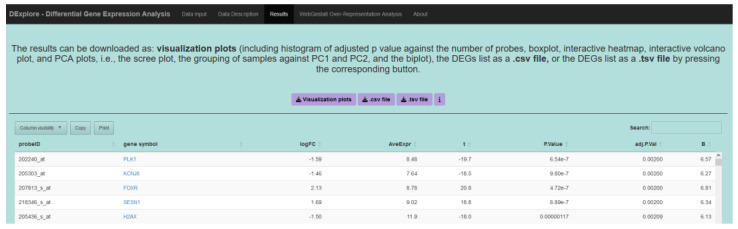
After analysis completion, DExplore presents a comprehensive list of differentially expressed genes (DEGs) in the ‘Results’ tab. Each entry includes the probe ID from Affymetrix, its corresponding gene symbol, and statistical values such as log_2_ fold-change, average expression, and *p*-value. Gene symbols are hyperlinked to NCBI’s Gene database for exploration. Users can also download a .zip file containing various graphical representations of the analysis, including heatmaps, volcano plots, histograms, boxplots, and PCA plots, facilitating further exploration and analysis of the DEGs list.

DExplore generates an annotated list of DEGs, providing both the probe ID used by Affymetrix and the corresponding gene symbol. Additionally, statistical values such as the log_2_ fold-change, average expression, and *p*-value are included for each gene (Figure 4). The gene symbol column also includes hyperlinks to NCBI’s Gene database, allowing users to further explore the listed genes. Furthermore, users can easily download a .zip file containing graphical representations of the differential expression analysis by clicking the corresponding button in the Results tab.

### 2.5. Functional Enrichment Analysis Using WebGestalt

After completing the differential expression analysis, the user can proceed directly to functional enrichment analysis of the identified DEGs. WebGestalt (WEB-based GEne SeT AnaLysis Toolkit) is one of the most widely used gene set enrichment analysis tools that helps users extract biological insights from the genes of interest. It was introduced in 2005 [55] and has been widely established among researchers for the interpretation of gene lists derived from large-scale omics studies, such as those from microarrays. WebGestalt can be freely accessed at https://www.webgestalt.org, accessed on 17 April 2024. In the most recent update of WebGestalt (2019), developers wrapped the core computing into an R package called WebGestaltR, which we used to provide users of DExplore with a direct connection to WebGestalt. 

By proceeding to the WebGestalt over-representation analysis using the corresponding tab panel, users may select one of the 12 organisms currently supported by WebGestalt and one of the reference sets for the selected organism to customize their analyses. The 12 organisms currently supported by WebGestalt are (1) *Arabidopsis thaliana* (athaliana), (2) *Bos taurus* (btaurus), (3) *Caenorhabditis elegans* (celegans), (4) *Canis lupus familiaris* (cfamiliaris), (5) *Danio rerio* (drerio), (6) *Sus scrofa* (sscrofa), (7) *Drosophila melanogaster* (dmelanogaster), (8) *Gallus gallus* (ggallus), (9) *Homo sapiens* (hsapiens), (10) *Mus musculus* (mmusculus), (11) *Rattus norvegicus* (rnorvegicus), and (12) *Saccharomyces cerevisiae* (scerevisiae). It is worth mentioning that DExplore’s utility for differential expression analysis is not confined to specific organisms, as it can analyze microarray data from all Affymetrix Gene Chips (deposited on GEO or uploaded by the user). However, the functional enrichment analysis with WebGestalt is limited to the aforementioned 12 organisms. DExplore via WebGestaltR renders the same output as that of the over-representation analysis using WebGestalt via its own website. The output can be viewed directly on the user’s browser and/or can be downloaded as a .zip file for saving or for further analyses. 

### 2.6. Docker Image

The introduction of a Docker version for DExplore offers several key advantages, emphasizing scalability and maintainability for our online analysis tool. Dockerization brings enhanced portability, isolation, scalability, versioning, resource efficiency, and consistency to the application [37,38]. Through encapsulation of the runtime environment into a single container, Docker ensures uniform behavior across diverse systems.

Versioning and rollbacks are simplified with Docker, allowing for easy tagging, and tracking of different releases. The isolation provided by Docker containers fosters a reproducible and predictable environment, thereby minimizing the likelihood of runtime errors. This approach not only enhances portability but also contributes to a consistent and reliable deployment process.

The Docker image provided through DExplore’s Data Input tab panel on Docker Hub is accessible for download and installation on any computer, facilitating local use in a manner identical to its online counterpart. This feature is particularly advantageous for users with poor internet connections or those wishing to upload their own data for a differential expression analysis. While the online version of DExplore supports data upload, it is strongly recommended to perform analyses locally, considering the 25 MB maximum size limit per uploaded file on the online version. A tutorial for running DExplore’s Docker version is available in Supplementary File S2.

### 2.7. Source Code Availability

DExplore can be found at www.dexplore.gr, accessed on 19 April 2024. The source code is available on GitHub (https://github.com/annakatsiki/dexplore, accessed on 19 April 2024) and the Docker image on the Docker Hub (https://hub.docker.com/r/akatsiki/dexplore, accessed on 19 April 2024). 

## 3. Results

In this section, we demonstrate the application of DExplore by analyzing two publicly available datasets and comparing our results with those obtained using GEO2R. We also conducted comparisons with CARMAweb, and the detailed results are provided in Supplementary File S3. Furthermore, we confirm the alignment of our findings with the existing literature.

### 3.1. Differential Expression Analysis

To assess the performance of our application, we utilized two datasets obtained from the GEO. 

#### 3.1.1. Dataset GSE39870

In the first dataset, MCF7 human breast cancer cells underwent a 3-day hormone-depletion period, followed by treatment with 10 μM doxorubicin for 12 h (accession number: GSE39870, Ref. [19]). The analysis was conducted using the Affymetrix GeneChip Human Genome U133A 2.0 Array, representing 14,500 well-characterized human genes. The comparative analysis involved MCF7 cells subjected to a vehicle treatment regimen versus those treated with 10 μM doxorubicin for 12 h. 

Applying criteria of absolute log_2_ fold-change ≥ 0.5 and adjusted *p*-value ≤ 0.05, with the false discovery rate (FDR) method [44] used for adjusting multiple comparisons, DExplore identified 739 differentially expressed genes. 

The same criteria were also applied to GEO2R [17], which is a well-established online tool from NCBI that is integrated into GEO to analyze datasets from the database. GEO2R identified 1055 differentially expressed genes. The full lists of the identified DEGs from both DExplore and GEO2R analyses, along with some statistical parameters, gene symbols, and gene names, are provided in the Supplementary Materials (Tables S1 and S2).

**Figure 5:**
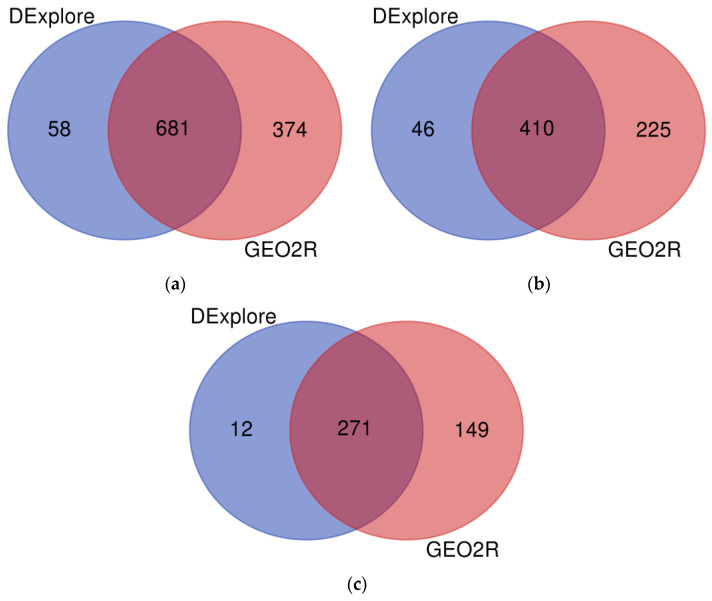
Venn diagram showing (**a**) all the differentially expressed genes, (**b**) the under-expressed genes, and (**c**) the over-expressed genes following treatment with doxorubicin identified by DExplore (light navy blue) and GEO2R (coral pink) (dataset GSE39870). To draw the diagrams, we used the “Venn Diagrams” online tool available at https://bioinformatics.psb.ugent.be/webtools/Venn/, accessed on 17 April 2024.

A total of 681 genes were identified as differentially expressed by both DExplore and GEO2R, comprising 410 down-regulated and 271 up-regulated genes (Figure 5). The complete list of common DEGs is available in Supplementary Materials (Tables S4–S6). 

**Figure 6:**
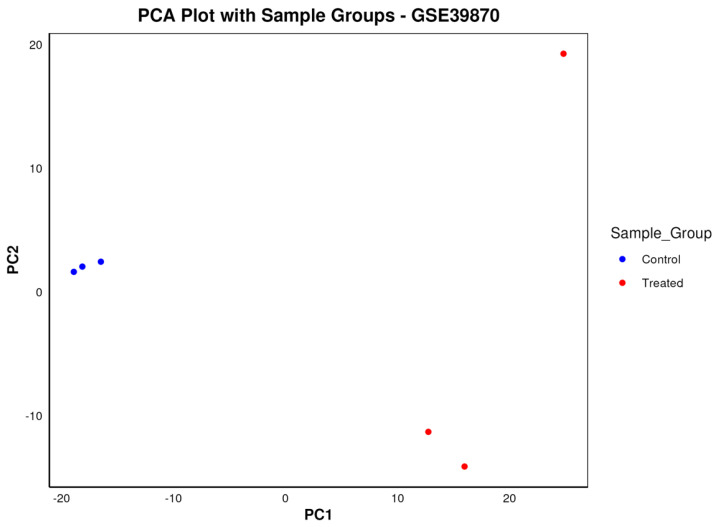
The PCA plot illustrates the results of principal component analysis for dataset GSE39870, presenting the six samples categorized into control and treated groups. Control samples are depicted in blue, while treated samples are colored red, facilitating visual discrimination between the two groups. Additionally, the plot showcases the distribution of the two groups in distinct regions of the PC1-PC2 two-dimensional space, providing insights into the separation and clustering patterns of the samples based on their gene expression profiles.

In addition to identifying differentially expressed genes, Dexplore also provides comprehensive visualizations of the dataset. Figure 6 illustrates the PCA results, showing the six samples in two groups, i.e., control and treated samples, against PC1 and PC2.

**Figure 7:**
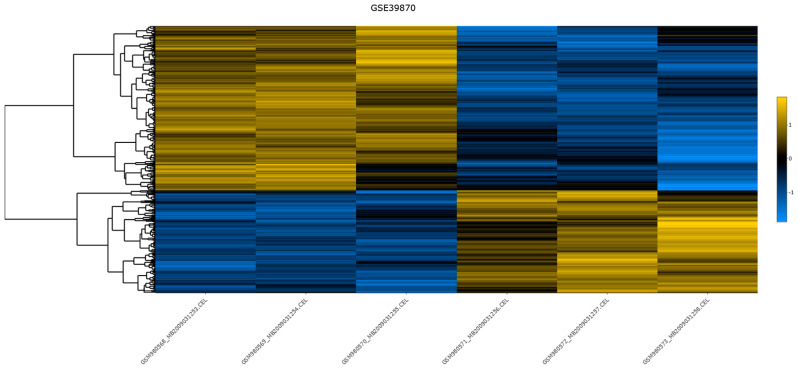
The heatmap for dataset GSE39870, generated by Dexplore. The produced dendrogram is displayed on the left side. For the clustering, Pearson correlation distance metric was utilized and row scaling was applied. The colors range from blue to yellow, reflecting the distance from the mean in units of standard deviation, since row scaling was applied, with blue colors corresponding to the negative values and yellows to the positive values. Additionally, the interactive version of this plot, available in Supplementary Materials (File S4), allows users to hover over each gene to view detailed information including the gene’s symbol, the sample name, the scaled value, and the adjusted *p*-value.

In Figure 7, we present a static image of the heatmap from the differential expression analysis, rendered by Dexplore. The interactive version of the heatmap, along with other plots rendered by Dexplore, is provided in Supplementary Materials (File S4).

**Figure 8:**
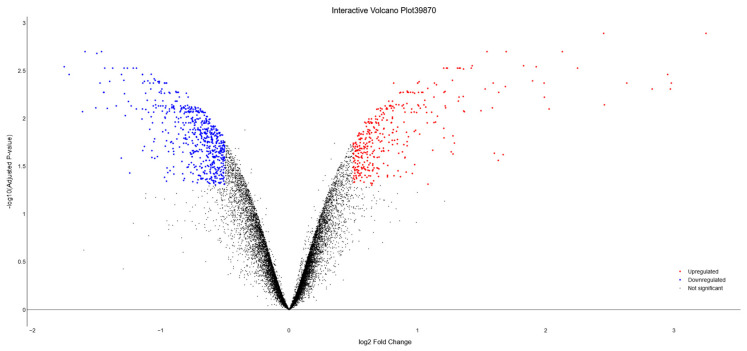
Volcano plot representing dataset GSE39870, generated by Dexplore. The plot visually highlights differentially expressed genes, with upregulated genes displayed in red, downregulated genes in blue, and genes with no significant or lower fold-change shown in black. Additionally, the interactive version of this plot, available in Supplementary Materials (File S4), allows users to hover over each gene to view detailed information including the gene’s symbol, log_2_FC, and adjusted *p*-value.

Figure 8 depicts a static image of the volcano plot, also rendered by Dexplore, where downregulated genes are shown in blue and upregulated ones in red. The interactive version of the volcano plot is also available in Supplementary Materials (File S4).

#### 3.1.2. Dataset GSE113427

The second dataset utilized to showcase Dexplore pertains to MDA-MB-231 human breast cancer cells. These cells underwent a 48 h pretreatment with a mixture of 40 μM oleic acid (OA) and 40 μΜ linoleic acid (LNA). Following the removal of the fatty acids, the cells were exposed to 0.41 μM doxorubicin for 24 h (accession number: GSE113427, [20,21]). The analysis utilized the Affymetrix GeneChip Human Genome 2.0 ST Array [transcript (gene) version], representing 26,000 well-characterized human genes. The comparative analysis involved MDA-MB-231 cells subjected to control treatment regimen versus those treated with 0.41 μM doxorubicin for 24 h.

The analysis was conducted using b”th D’xplore and GEO2R, applying the same criteria as described for dataset GSE39870. Dexplore identified 237 differentially expressed genes, while GEO2R identified 243 differentially expressed genes. The complete lists of the identified DEGs from both Dexplore and GEO2R analyses, along with statistical parameters, gene symbols, and gene names, can be found in Supplementary Materials (Tables S25 and S26).

**Figure 9:**
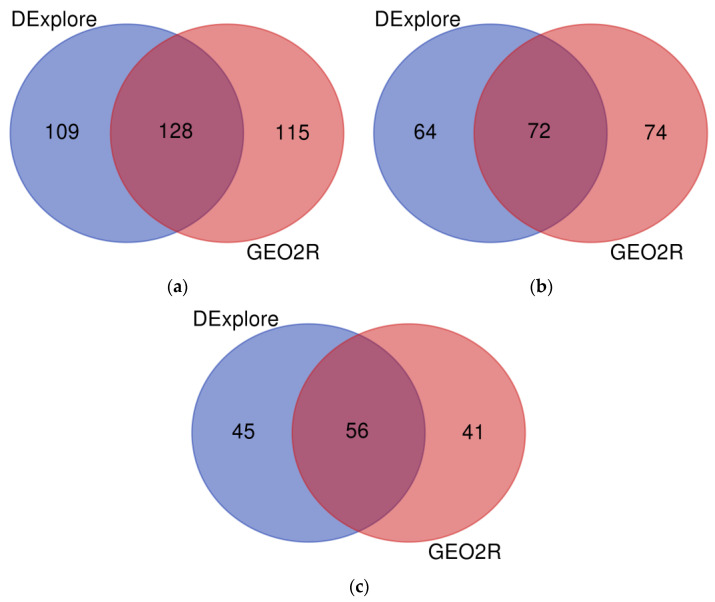
Venn diagram showing (**a**) all the differentially expressed genes, (**b**) the under-expressed genes, and (**c**) the over-expressed genes following treatment with doxorubicin identified by DExplore (light navy blue) and GEO2R (coral pink) (dataset GSE113427). To draw the diagrams, we used the “Venn Diagrams” online tool available at https://bioinformatics.psb.ugent.be/webtools/Venn/, accessed on 17 April 2024.

A total of 128 genes were identified as differentially expressed by both Dexplore and GEO2R, comprising 72 down-regulated and 56 up-regulated genes (Figure 9). The complete list of common DEGs is available in Supplementary Materials (Tables S28–S30). 

**Figure 10:**
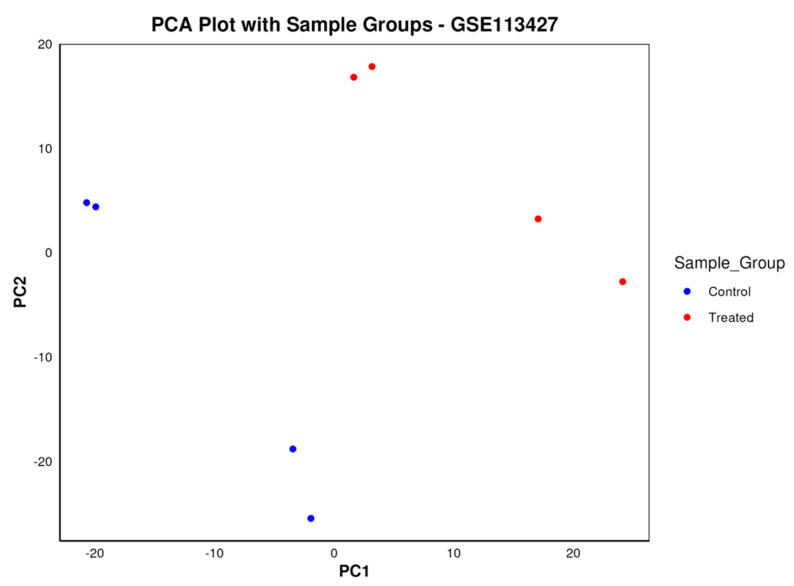
The PCA plot illustrates the results of principal component analysis for dataset GSE113427, presenting the eight samples that were compared categorized into control and treated groups. Control samples are depicted in blue, while treated samples are colored red, facilitating visual discrimination between the two groups. Additionally, the plot showcases the distribution of the two groups in distinct regions of the PC1-PC2 two-dimensional space, providing insights into the separation and clustering patterns of the samples based on their gene expression profiles.

Figure 10 presents the PCA plot, illustrating the grouping of samples against PC1 and PC2. 

**Figure 11:**
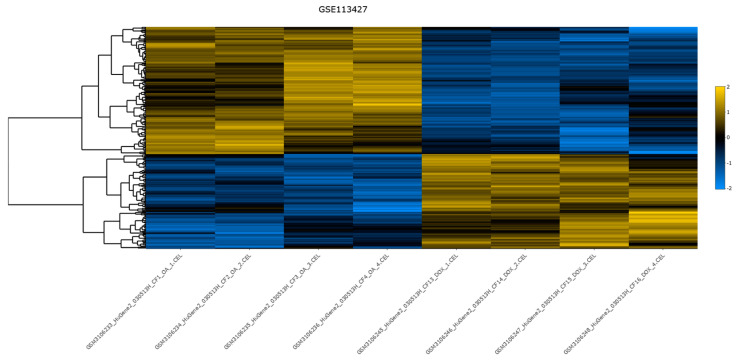
The heatmap for dataset GSE113427, generated by DExplore. Only the samples of interest are shown. The produced dendrogram is displayed on the left side. For the clustering, Pearson correlation distance metric was utilized, and row scaling was applied. The colors range from blue to yellow, reflecting the distance from the mean in units of standard deviation, since row scaling was applied, with blue colors corresponding the negative values and yellows the positive values. Additionally, the interactive version of this plot, available in Supplementary Materials (File S4), allows users to hover over each gene to view detailed information including the gene’s symbol, the sample name, the scaled value, and the adjusted *p*-value.

In Figure 11, a static heatmap from the differential expression analysis is presented, offering insights into the expression patterns of differentially expressed genes. 

**Figure 12:**
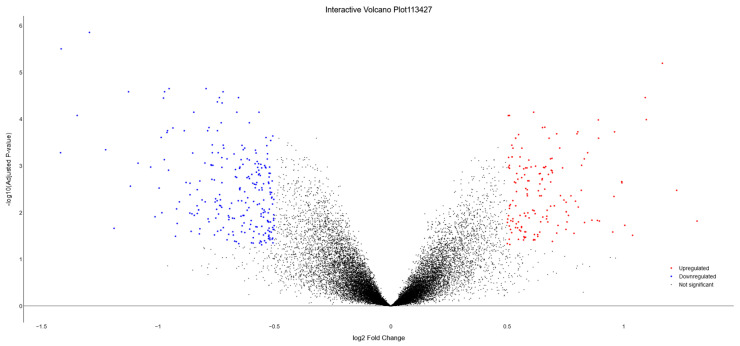
Volcano plot representing dataset GSE113427, generated by DExplore. The plot visually highlights differentially expressed genes, with upregulated genes displayed in red, downregulated genes in blue, and genes with no significant or lower fold-change shown in black. Additionally, the interactive version of this plot, available in Supplementary Materials (File S5), allows users to hover over each gene to view detailed information including the gene’s symbol, log_2_FC, and adjusted *p*-value.

Figure 12 showcases a static volcano plot, highlighting downregulated genes in blue and upregulated genes in red. The interactive versions of both these plots, along with others rendered by DExplore for this dataset, can be found in Supplementary Materials (File S5).

### 3.2. Functional Enrichment Analysis Using WebGestalt

To gain biological insights into the functional roles of the identified DEGs, we performed the WebGestalt ORA using the results from both DExplore and GEO2R. This analysis, seamlessly integrated into our application, facilitates the identification of enriched gene ontology (GO) terms within a subset of genes.

**Figure 13:**
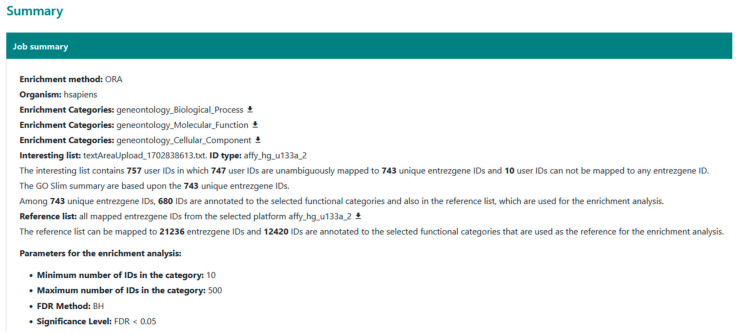
The parameters used for WebGestalt over-representation analysis (ORA) of dataset GSE39870.

The parameters employed for WebGestalt’s ORA are illustrated in Figure 13. For the input list, we directly uploaded the probe IDs of the identified DEGs to prevent any potential confusion caused by unannotated or inaccurately annotated probes. Accordingly, the ID type used was affy_hg_u133a_2 for dataset GSE39870 and affy_hugene_2_0_st_v_1 for dataset GSE113427, corresponding to the probe IDs. The analysis was conducted individually for under- and over-expressed genes identified by both DExplore and GEO2R, as well as for all DEGs. 

#### 3.2.1. Dataset GSE39870

**Figure 14:**
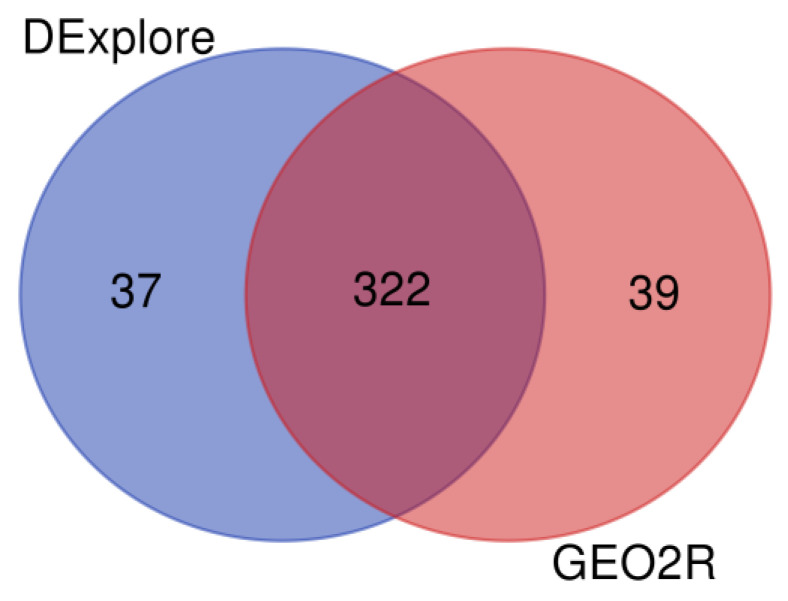
Venn diagram showing the comparison of enriched Gene Ontology terms derived from WebGestalt over-representation analysis (ORA) using all differentially expressed genes detected by DExplore (light navy blue) and GEO2R (coral pink) for dataset GSE39870. To draw the diagrams, we used the “Venn Diagrams” online tool available at https://bioinformatics.psb.ugent.be/webtools/Venn/, accessed on 17 April 2024.

The WebGestalt’s ORA for dataset GSE39870 identified 359 enriched GO terms using all DEGs detected with DExplore. The same input and statistical parameters were used for the analysis with GEO2R and resulted in 361 enriched GO terms. As depicted in Figure 14, both analyses shared 322 common enriched GO terms (Table S10). Detailed results, along with comparisons between DExplore and GEO2R, considering separately under- or over-expressed DEGs, are available in Supplementary Materials (Tables S10–S24).

#### 3.2.2. Dataset GSE113427

**Figure 15:**
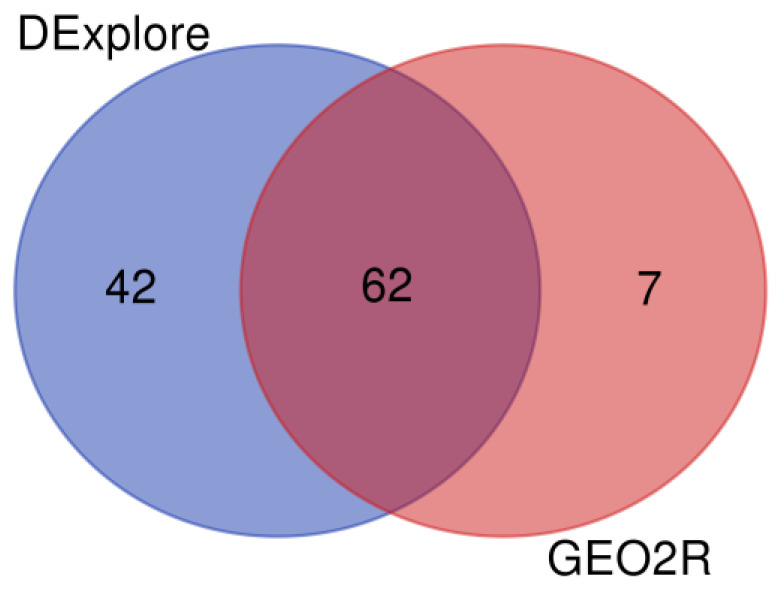
Venn diagram showing the comparison of enriched Gene Ontology terms derived from WebGestalt over-representation analysis (ORA) using all differentially expressed genes detected by DExplore (light navy blue) and GEO2R (coral pink) for dataset GSE113427. To draw the diagrams, we used the “Venn Diagrams” online tool available at https://bioinformatics.psb.ugent.be/webtools/Venn/, accessed on 17 April 2024.

The WebGestalt’s ORA for dataset GSE113427 revealed 104 enriched GO terms when using all DEGs detected with DExplore. Employing the same input and statistical parameters for the analysis with GEO2R resulted in 69 enriched GO terms. Figure 15 illustrates that both analyses shared 62 common enriched GO terms (Table S43). Additional comprehensive results, including comparisons between DExplore and GEO2R and considering separately under- or over-expressed DEGs, are available in Supplementary Materials (Tables S34–S47).

### 3.3. Comparative Analysis of DExplore against GEO2R

To evaluate DExplore’s performance against GEO2R, we assessed the concordance between their identified differentially expressed genes (DEGs) and enriched gene ontology (GO) terms using WebGestalt for a functional enrichment analysis. The overlap ratio, measuring the proportion of common DEGs identified by both methods relative to the total number of DEGs identified by either method, was computed. Additionally, we calculated the false discovery rate (FDR), representing the proportion of DEGs uniquely identified by one application relative to the total number of DEGs identified by that application. A lower FDR indicates higher confidence in the significance of the identified features.

For dataset GSE39870, DExplore exhibited an overlap ratio of 0.612, whereas GEO2R showed a higher ratio of 0.948, indicative of GEO2R identifying more DEGs in this dataset. However, when comparing the overlap ratio of enriched GO terms, where the number of terms is similar, DExplore showed a notable increase to 0.81, representing a substantial 20% improvement. This suggests that DExplore’s functional enrichment analysis provides enhanced detection of the affected biological processes in response to doxorubicin treatment.

Similarly, for dataset GSE113427, DExplore yields an overlap ratio of 0.364 for DEGs, while GEO2R exhibits a higher ratio of 0.690, reflecting the larger number of DEGs identified by GEO2R. However, the overlap ratio of enriched GO terms increases to 0.559 for DExplore, marking a significant 20% increase and indicating improved detection of underlying biological processes compared to GEO2R.

Furthermore, DExplore demonstrates a significantly lower FDR of 0.079 for all identified DEGs in GSE39870, compared to GEO2R’s FDR of 0.355. Similarly, in GSE113427, DExplore’s FDR of 0.460 is comparable to GEO2R’s FDR of 0.473. These findings highlight DExplore’s effectiveness in analyzing high-throughput gene expression data.

It is noteworthy that DExplore’s approach to identifying DEGs emphasizes specificity, which could explain the smaller set of DEGs compared to GEO2R. This conservative approach tends to reduce false positives, leading to a more reliable set of high-confidence DEGs for downstream analysis. However, this comes with the trade-off of potentially increasing the false negative rate, as some true positives might not be detected due to more stringent criteria.

Despite this trade-off, the lower FDR and improved GO term overlap ratios suggest that DExplore’s conservative approach yields a high-quality set of DEGs, providing a robust foundation for downstream functional enrichment analysis. This balance between reducing false positives and minimizing false negatives reflects our commitment to providing users with reliable and accurate results while acknowledging the inherent uncertainties in gene expression analysis.

### 3.4. Summarization and Visualization Using REVIGO

To deepen our understanding of the results of the WebGestalt’s ORA, we took advantage of REVIGO [35]. REVIGO, accessible at http://revigo.irb.hr/ (accessed on 28 February 2024), is a web server designed to summarize and visualize extensive lists of GO terms. This computational tool accomplishes two primary tasks: (a) it condenses lengthy GO lists by reducing functional redundancies through semantic similarity; and (b) it presents the remaining GO terms using two-dimensional plots, interactive graphs, tree-maps, or tag clouds.

**Figure 16:**
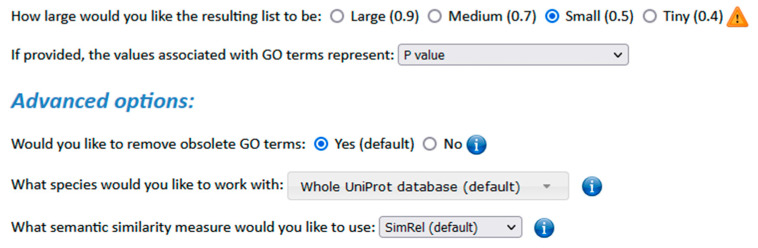
The parameters chosen for REVIGO analysis.

As input for REVIGO, we provided the GO terms and their corresponding FDR values, which were derived from the WebGestalt’s ORA for all DEGs detected by DExplore. The chosen parameters are illustrated in Figure 16. The output from REVIGO, including representative GO terms for each cluster, are presented in distinct sets of tables for the two analyzed datasets. These tables correspond to the Biological Process, Cellular Component, and Molecular Function categories for both dataset GSE39870 and dataset GSE113427. Table 1, Table 2 and Table 3 associated with dataset GSE39870 cover aspects of Biological Process, Cellular Component, and Molecular Function, while Table 4, Table 5 and Table 6 related to dataset GSE113427 provide additional insights into these categories.

DEGs obtained through DExplore and the enriched GO terms from the subsequent functional enrichment analysis using the ORA in WebGestalt reveal dysregulation in key biological processes following the treatment of MCF7 human breast cancer cells with doxorubicin. Specifically, we observe dysregulation in the DNA replication process (e.g., GO:0006260—DNA replication, GO:0006301—post-replication repair, GO:0022616—DNA strand elongation, and GO:0051095—regulation of helicase activity) and disruption of cell cycle progression (e.g., GO:0000280—nuclear division, GO:0007077—mitotic nuclear membrane disassembly, GO:0031145—anaphase-promoting complex-dependent catabolic process, GO:0032886—regulation of microtubule-based process, GO:0044770—cell cycle phase transition, GO:0050000—chromosome localization, GO:0051301—cell division, GO:0051302—regulation of cell division, GO:0051726—regulation of cell cycle, GO:0051782—negative regulation of cell division, GO:0051783—regulation of nuclear division, and GO:1901987—regulation of cell cycle phase transition). Furthermore, we detect changes due to a reactive nitrogen species stimulus (GO: 1902170—cellular response to reactive nitrogen species) and dysfunction in iron homeostasis (GO:0006879—intracellular iron ion homeostasis).

The results for dataset GSE113427, involving the treatment of MDA-MB-231 human breast cancer cells with doxorubicin, exhibited similar patterns. However, due to the smaller number of identified DEGs, the subsequent functional enrichment analysis using the ORA in WebGestalt resulted in a reduced number of enriched GO terms. Nevertheless, it is evident that there is dysregulation in mitotic nuclear division, as indicated by GO:0007088—regulation of mitotic nuclear division, GO:0031145—anaphase-promoting complex-dependent catabolic process, GO:0032886—regulation of microtubule-based process, GO:0050000—chromosome localization, and GO:0140014—mitotic nuclear division.

The observed differences can be attributed to inherent molecular distinctions between the two distinct breast cancer cell lines; for instance, MCF7 cells are ER^+^ (estrogen receptor +), whereas MDA-MB-231 cells are ER^-^ (estrogen receptor −). Additionally, variations in experimental protocols, including differences in doxorubicin concentration (10 μM vs. 41 μM) and treatment duration (12 h vs. 24 h), contribute to the variations in the results.

## 4. Discussion

Differential gene expression analysis is a routine task for researchers investigating the impact of various treatments on cell lines or tissues. Despite the emergence of RNA-sequencing as a viable alternative, the microarray technology remains a popular choice for its cost-effectiveness, high-throughput, and the substantial volume of data accumulated over more than 15 years, cementing microarrays as a robust and established method [3,4,5,6].

Given the continued use of microarrays, numerous online tools and local applications have been developed to identify DEGs from microarray experiments. However, many of these tools are no longer maintained, and others either require payment or demand programming expertise [9,10,11,12,13,14,56]. To address these challenges, we introduced DExplore. It is an online application designed to detect DEGs between two experimental conditions using data from the Affymetrix GeneChips available on NCBI GEO or stored locally, without the need for programming skills.

It is common knowledge that there is an inherent challenge in the field of differential expression analysis [37,53,54,57]. Since the exact gene expressions are not known, identifying DEGs between two experimental conditions lacks a single “gold” standard tool [53,58,59,60,61]. Consequently, making comparisons among available applications is challenging and can be misleading. Nevertheless, the findings of differential expression analysis with DExplore are in good accordance with those from GEO2R, a well-established and widely recognized online tool dedicated to differential expression analysis, exclusively utilizing data from the NCBI GEO. The minor differences observed in the showcased examples can be mitigated through the performance of functional enrichment analysis. 

Doxorubicin, formerly known as Adriamycin, is a cytotoxic anthracycline antibiotic isolated from cultures of *Streptomyces peucetius* var. *caesius*. It is used as a chemotherapeutic drug to treat various malignancies, including leukemias, lymphomas, metastatic breast cancer, ovarian carcinomas, soft tissue, and bone sarcomas, as described in the FDA’s Drug database (https://www.accessdata.fda.gov/scripts/cder/daf/, accessed on 22 January 2024). 

There are mainly three proposed mechanisms for doxorubicin’s anticancer properties: DNA intercalation, topoisomerase II inhibition, and free radical generation, leading to the induction of regulated cell death [62,63,64]. Doxorubicin and the related anthracyclines consist of flat aromatic moieties that intercalate between DNA bases, each anchored tightly by one or more sugars in the minor groove. Intercalation pushes apart the neighboring bases, resulting in the bidirectional transmission of positive torsion. These alterations in DNA structure can inhibit enzymes, including topoisomerases [65]. Doxorubicin can also trap topoisomerase II in the double-strand cleavage form and prevent ligation, as it stabilizes the DNA-topoisomerase II cleavage complex [63]. The generation of free radicals by doxorubicin causes damage to cellular membranes, DNA, and proteins [66]. Specifically, it has been shown that doxorubicin causes lipid peroxidation, membrane damage, and DNA damage oxidative stress [66] and also triggers various pathways of cell death, including apoptosis, autophagy, necroptosis, and ferroptosis [62]. 

The results from DExplore and the subsequent functional enrichment analysis using the ORA with WebGestalt not only corroborate existing findings but also unveil additional specific biological insights into the mechanism by which doxorubicin exerts its anticancer activity. Specifically, dysregulation in the DNA replication process and disruption of cell cycle progression suggest potential disturbances caused by DNA intercalation induced by doxorubicin. Furthermore, our results support the proposed mechanism whereby doxorubicin contributes to the production of intracellular nitrogen free radicals, as indicated by GO:190270—cellular response to reactive nitrogen species. We also detect dysfunction in iron homeostasis (GO:0006879—intracellular iron ion homeostasis), likely linked to the mechanism of programmed cell death via ferroptosis.

Comparing our results to the publications associated with the case study datasets, common findings emerge. However, the use of DExplore and WebGestalt’s ORA detected additional biological processes. Specifically, in the original publication for dataset GSE39870 [19], cell cycle dysfunctions and apoptosis were identified, aligning with our results, but nitrosative stress and iron ion homeostasis dysregulations were not mentioned. It is worth noting that in the original paper, a different, more stringent threshold in the *p*-value (*p*-value < 0.01) was used, with no reference to fold-change threshold. As for dataset GSE113427, the original publication [21] used a cutoff of *p*-value < 0.05, similar to our approach, but with a stricter log_2_ fold change threshold (1.2). Their analysis, like ours, detected differentially expressed genes involved in cell death, apoptosis, cell survival, and cell cycle regulation. 

Our comparative analysis of DExplore alongside GEO2R and CARMAweb revealed insights into their performance in identifying DEGs and enriched GO terms. Initially, both GEO2R and CARMAweb identified a larger number of DEGs compared to DExplore across the GSE39870 and GSE113427 datasets. However, further analysis, including overlap ratio and false discovery rate (FDR) calculations, shed light on the effectiveness of DExplore’s approach. Notably, after subjecting the data to gene enrichment analysis, we observed a significant reduction in the initially observed disparity. In both showcased datasets, the overlap ratio increased by approximately 20% following functional enrichment analysis. This enhancement underscores the value of DExplore’s integrated functional enrichment analysis, which provides deeper insights into the underlying biological processes beyond the sheer number of identified DEGs. 

Additionally, DExplore exhibited a significantly lower FDR compared to GEO2R and CARMAweb, indicating higher confidence in the identified DEGs. These findings suggest that DExplore’s conservative approach tends to reduce false positives, providing a more reliable set of high-confidence DEGs. This reduced false positive rate can improve downstream analyses, such as Gene Ontology (GO) term enrichment, leading to more accurate interpretations of biological processes and pathways.

However, this approach may increase the false negative rate, implying that some true positive DEGs could be missed due to stringent criteria. While this trade-off prioritizes specificity and reliability, researchers should be aware of this balance when choosing tools for gene expression analysis.

Overall, these findings highlight the robustness and reliability of DExplore in analyzing high-throughput gene expression data, positioning it as a valuable tool for uncovering biologically relevant insights from microarray experiments. Future studies may explore strategies to balance specificity and sensitivity in DEG identification to ensure comprehensive results.

The integration of functional enrichment analysis as a complementary step to differential expression analysis proves to be crucial, especially given the absence of a gold standard for microarray differential expression analysis. Designed for seamless integration, DExplore, with its connection to WebGestalt, provides researchers with a comprehensive view of the biological processes and molecular functions associated with the identified differentially expressed genes.

Delving into a detailed exploration of the disparities observed in DE gene analysis among DExplore, GEO2R, and CARMAweb, multiple factors come into play. Firstly, variations in R and Bioconductor package versions may play a role. For instance, different versions of the limma package are utilized: 3.26.9 in CARMAweb, 3.54.0 in GEO2R, and 3.58.1 in DExplore. This difference in package versions could potentially contribute to the observed divergent outcomes.

Furthermore, the preprocessing methods applied by each tool introduce another layer of diversity. CARMAweb utilizes the rma function of the affy package [67], GEO2R performs log_2_ transformation and normalization using the normalizeBetweenArrays function of the limma package [27], while DExplore employs the rma function of the oligo package [26], encompassing background correction, normalization, and summarization. Existing literature emphasizes that the choice of preprocessing algorithm significantly influences analysis outcomes [59].

An additional point of divergence lies in the annotation process [37,60,68]. DExplore employs the annotate Bioconductor package [28], utilizing platform-specific design files for microarray chips. In contrast, GEO2R and CARMAweb lack explicit disclosure regarding the annotation package used, introducing an element of ambiguity.

Additionally, the absence of access to individual results at each step of the analysis complicates the pinpointing of the exact divergence point. Regardless of the specific genes identified as differentially expressed, our results underscore the reconciling impact of functional enrichment analysis. As demonstrated in the two case studies, the incorporation of this additional step not only diminishes discrepancies but also reveals converging biological insights, as evidenced by the noTable 20% increase observed in the overlap ratio after applying the functional enrichment analysis.

This multifaceted analysis underscores the complexity of DE gene analysis, urging a nuanced interpretation of differences and highlighting the importance of comprehensive methodological transparency.

Moreover, one of the notable strengths of DExplore lies in its versatility. It is not confined to working solely with .CEL files submitted to NCBI GEO; instead, it offers flexibility by accommodating uploaded data, facilitating both online usage and local application. This flexibility is further enhanced by the availability of a Docker image on Docker Hub, enabling users to seamlessly incorporate DExplore into their analyses in diverse computing environments.

Furthermore, DExplore stands out from other applications like CARMAweb and GEO2R by providing comprehensive visualization plots, including interactive options such as heatmaps and volcano plots. Unlike these alternatives, DExplore’s plots offer enhanced interactivity, providing users with a dynamic and informative exploration of their data. This feature sets DExplore apart, offering users a more intuitive and insightful analysis experience.

## 5. Conclusions

In summary, we introduce an open-source, robust application designed to serve as a reference, simplifying the intricacies of publicly available high-throughput data. DExplore (www.dexplore.gr, accessed on 19 April 2024) aims to assist molecular biologists in focusing on the select few genes that hold biological relevance to their experiments. Whether utilized online or locally, DExplore offers flexibility, and a Docker image is conveniently available through our website. Additionally, users can seamlessly conduct functional enrichment analysis using the popular online tool WebGestalt directly within the DExplore website. Furthermore, DExplore stands out by providing comprehensive visualization plots, including interactive options such as heatmaps and volcano plots. These plots offer enhanced interactivity, providing users with a dynamic and informative exploration of their data.

As mentioned before, DExplore performs comparably to GEO2R, a well-established online tool for differential expression analysis using data available in the NCBI GEO. In the showcased examples, GEO2R identified almost the same number or more genes as differentially expressed than DExplore, but this difference appears to be overcome when gene enrichment analysis is applied. Notably, functional enrichment analysis with the integrated WebGestalt led to a significant increase in the overlap ratio of identified differentially expressed genes, indicating the enhanced detection of affected biological processes. Furthermore, DExplore exhibited a significantly lower false discovery rate (FDR) for all identified DEGs compared to GEO2R, further underscoring its reliability in identifying differentially expressed genes with higher confidence. Additionally, the results obtained using DExplore, followed by functional enrichment analysis with the integrated WebGestalt, align with the known mechanism through which doxorubicin exerts its anticancer activity. Moreover, DExplore offers versatility, allowing usage not only with .CEL files submitted to the NCBI GEO but also with uploaded data, online, and locally, due to the Docker image available on Docker Hub.

DExplore currently has a limitation, restricting its analysis to raw data from microarray experiments utilizing Affymetrix Gene Chips. While this limitation is acknowledged, we are committed to addressing it in future releases. Additionally, we plan to integrate gene set enrichment analysis (GSEA), a second-generation pathway analysis, into DExplore to enhance the functional characterization of differentially expressed genes.

The source code is available on GitHub (https://github.com/annakatsiki/dexplore, accessed on 19 April 2024) and the Docker image on the Docker Hub (https://hub.docker.com/r/akatsiki/dexplore, accessed on 19 April 2024).

## Figures and Tables

**Table 1:** The results of REVIGO for Gene Ontology (GO) Biological Process for dataset GSE39870. Only the representative GO terms for each identified cluster are displayed here.

Term ID	Name
GO:0000280	nuclear division
GO:0002483	antigen processing and presentation of endogenous peptide antigen
GO:0006260	DNA replication
GO:0006301	post replication repair
GO:0006403	RNA localization
GO:0006596	polyamine biosynthetic process
GO:0006760	folic acid-containing compound metabolic process
GO:0006879	intracellular iron ion homeostasis
GO:0006913	nucleocytoplasmic transport
GO:0007077	mitotic nuclear membrane disassembly
GO:0008630	intrinsic apoptotic signaling pathway in response to DNA damage
GO:0009314	response to radiation
GO:0010839	negative regulation of keratinocyte proliferation
GO:0022616	DNA strand elongation
GO:0030330	DNA damage response, signal transduction by p53 class mediator
GO:0031099	Regeneration
GO:0031100	animal organ regeneration
GO:0031123	RNA 3′-end processing
GO:0031124	mRNA 3′-end processing
GO:0031145	anaphase-promoting complex-dependent catabolic process
GO:0031503	protein-containing complex localization
GO:0032886	regulation of microtubule-based process
GO:0032922	circadian regulation of gene expression
GO:0034502	protein localization to chromosome
GO:0038111	interleukin-7-mediated signaling pathway
GO:0042594	response to starvation
GO:0042770	signal transduction in response to DNA damage
GO:0043484	regulation of RNA splicing
GO:0044770	cell cycle phase transition
GO:0046040	IMP metabolic process
GO:0050000	chromosome localization
GO:0050657	nucleic acid transport
GO:0051052	regulation of DNA metabolic process
GO:0051054	positive regulation of DNA metabolic process
GO:0051095	regulation of helicase activity
GO:0051301	cell division
GO:0051302	regulation of cell division
GO:0051642	centrosome localization
GO:0051726	regulation of cell cycle
GO:0051782	negative regulation of cell division
GO:0051783	regulation of nuclear division
GO:0060576	intestinal epithelial cell development
GO:0061351	neural precursor cell proliferation
GO:0061842	microtubule organizing center localization
GO:0070734	histone H3-K27 methylation
GO:0071166	ribonucleoprotein complex localization
GO:0071824	protein-DNA complex organization
GO:0072001	renal system development
GO:0072331	signal transduction by p53 class mediator
GO:0104004	cellular response to environmental stimulus
GO:1901987	regulation of cell cycle phase transition
GO:1902170	cellular response to reactive nitrogen species
GO:1903311	regulation of mRNA metabolic process
GO:2000241	regulation of reproductive process
GO:2000736	regulation of stem cell differentiation
GO:2000241	regulation of reproductive process
GO:2000736	regulation of stem cell differentiation

**Table 2:** The results of REVIGO for Gene Ontology (GO) Cellular Component for dataset GSE39870. Only the representative GO terms for each identified cluster are presented here.

Term ID	Name
GO:0000307	cyclin-dependent protein kinase holoenzyme complex
GO:0000793	condensed chromosome
GO:0005635	nuclear envelope
GO:0005819	Spindle
GO:0030496	Midbody
GO:0032300	mismatch repair complex
GO:0032993	protein-DNA complex
GO:0090543	Flemming body

**Table 3:** The results of REVIGO for Gene Ontology (GO) Molecular Function for dataset GSE39870. Only the representative GO terms for each identified cluster are displayed here.

Term ID	Name
GO:0000217	DNA secondary structure binding
GO:0003682	chromatin binding
GO:0003684	damaged DNA binding
GO:0003688	DNA replication origin binding
GO:0003697	single-stranded DNA binding
GO:0003777	microtubule motor activity
GO:0004674	protein serine/threonine kinase activity
GO:0005515	protein binding
GO:0008094	ATP-dependent activity, acting on DNA
GO:0015631	tubulin binding
GO:0016791	phosphatase activity
GO:0019887	protein kinase regulator activity
GO:0044389	ubiquitin-like protein ligase binding
GO:0140297	DNA-binding transcription factor binding

**Table 4:** The results of REVIGO for Gene Ontology (GO) Biological Process for dataset GSE113427. Only the representative GO terms for each identified cluster are displayed here.

Term ID	Name
GO:0007088	regulation of mitotic nuclear division
GO:0007163	establishment or maintenance of cell polarity
GO:0030010	establishment of cell polarity
GO:0031145	anaphase-promoting complex-dependent catabolic process
GO:0032886	regulation of microtubule-based process
GO:0048285	organelle fission
GO:0050000	chromosome localization
GO:0050909	sensory perception of taste
GO:0140014	mitotic nuclear division

**Table 5:** The results of REVIGO for Gene Ontology (GO) Cellular Component for dataset GSE113427. Only the representative GO terms for each identified cluster are presented here.

Term ID	Name
GO:0000775	chromosome, centromeric region
GO:0005819	spindle
GO:0030496	midbody

**Table 6:** The results of REVIGO for Gene Ontology (GO) Molecular Function for dataset GSE113427. Only the representative GO terms for each identified cluster are displayed here.

Term ID	Name
GO:0008017	microtubule binding
GO:0008527	taste receptor activity

## Data Availability

Data used to exemplify the usage of the tool can be accessed on GEO with accession numbers GSE39870 and GSE113427. DExplore is available as a web service through www.dexplore.gr (accessed on 19 April 2024). The source code is publicly available for download at https://github.com/annakatsiki/dexplore (accessed on 19 April 2024), and the Docker image is available on the Docker Hub (https://hub.docker.com/r/akatsiki/dexplore) (accessed on 19 April 2024).

## Supplementary Materials

The following supporting information can be downloaded at https://www.mdpi.com/article/10.3390/biology13050351/s1; File S1: DExplore’s User’s Guide; File S2: Tutorial for DExplore’s Docker version; File S3: DExplore’s comparison to CARMAweb using datasets GSE39870 and GSE113427; File S4: Zip file with the visualization plots from DExplore’s analysis using dataset GSE39870; File S5: Zip file with the visualization plots from DExplore’s analysis using dataset GSE113427; Tables S1–S9: Lists of DEGs detected by DExplore, GEO2R, and CARMAweb using dataset GSE39870. Tables S10–S24: Lists of GO terms identified by WebGestalt’s ORA using DEGs detected by DExplore, GEO2R, and CARMAweb using dataset GSE39870, Tables S25–S33: Lists of DEGs detected by DExplore, GEO2R, and CARMAweb using dataset GSE113427; Tables S34–S47: Lists of GO terms identified by WebGestalt’s ORA using DEGs detected by DExplore, GEO2R, and CARMAweb using dataset GSE113427. References [16,18,19,20,21,27,41,42,43,44,45,46,47,51,69,70,71,72,73] are cited in the supplementary materials.
