# Supplementary material for: DExplore: An Online Tool for Detecting Differentially Expressed Genes from mRNA Microarray Experiments
Source: Biology (Basel). 2024 May 16;13(5):351. doi: 10.3390/biology13050351 (PMC11117493; doi:10.3390/biology13050351)
Supplement: Supplementary file 1 [file biology-13-00351-s001.zip › S1. DExplore_User's_Guide.pdf]

DExplore - Differential Gene Expression Analysis

# DExplore- User's guide

## 1. Introduction

DExplore is an online and user-friendly tool designed for detecting differentially expressed (DE) genes using data sourced from the international public repository NCBI GEO. In addition to identifying DE genes, DExplore also provides interactive graphical representations, including heatmaps and volcano plots. This feature enables users to easily visualize the data. Complementing histograms, boxplots, and PCA plots are also provided. Furthermore, DExplore offers the capability to perform functional enrichment analysis using a built-in version of the well-established web tool WebGestalt[1] (www.webgestalt.org).

Users can also upload data that have not been submitted to GEO yet. These user-uploaded data are only temporarily saved on the server and are automatically deleted as soon as the user exits the platform, or their session expires. This ensures that there is no danger of the data being copied or used by anyone other than the user.

DExplore is built utilizing the R programming language and Bioconductor and can be easily used by researchers with no specialized programming skills require.

The user interface is quite simple, and the application runs exclusively online, so users do not have to download or store raw data in their computer.

In addition, both the source code and the docker image can be accessed and downloaded using the links on the homepage.

Currently, DExplore can only be used to analyze single-channel mRNA microarray experiments for Affymetrix® platforms but will be expanded soon to be used for other commercially available platforms such as Illumina and Agilent.

## 2. How to use

DExplore is comprised of four tab panels; Data Input, Data Description, Results and WebGestalt Over-Representation Analysis.

Do not forget to refresh DExplore between analyses.

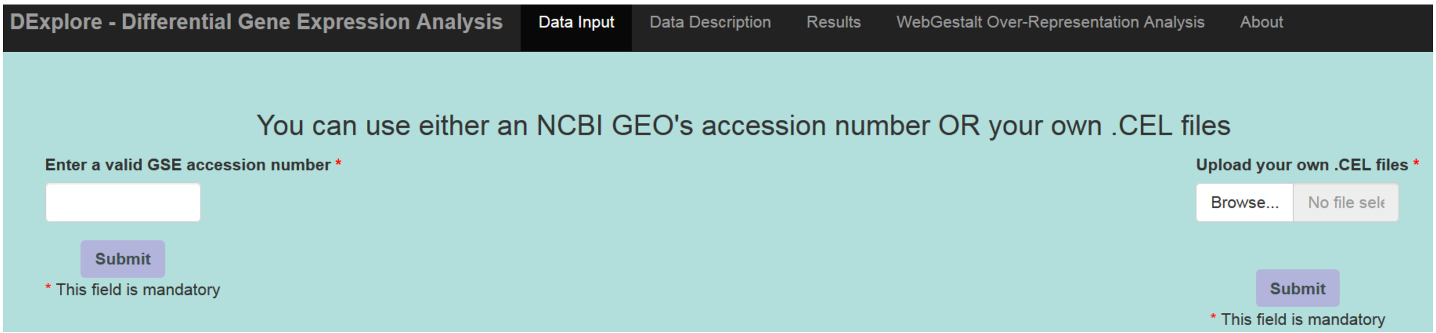


Analyses differ depending on the type of data the user wants to use.

### a. Using DExplore with NCBI GEO's microarray data

The first thing users must do is to enter a valid GSE accession number from the NCBI GEO Database (https://www.ncbi.nlm.nih.gov/geo/) and press the button "Submit" in the "Data Input" tab.

Important notice: enter only the number without writing "GSE" (e.g., for Series "GSE41827", enter "41827").

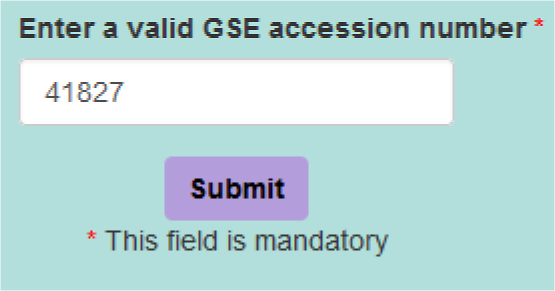


After a few minutes (time depends on the submitted data size), DExplore provides a hyperlink to the GEO's corresponding page.

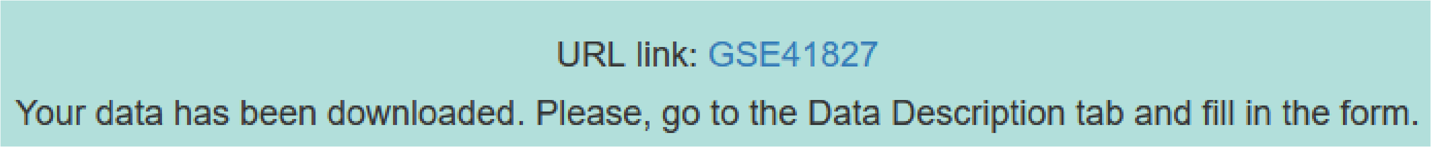


In the Data Description tab, users must choose the parameters for analysis, including the platform to be used (important for experiments utilizing microarray chips from multiple platforms), the comparison criterion, and which samples are to be treated as controls and compared to. After selecting each parameter, users should press the submit button to proceed.

Subsequently, the user must select certain statistical parameters required for the analysis, including the method for adjusting the p-value for multiple comparisons, the absolute $\log_2$ Fold Change threshold, and the adjusted p-value threshold (see Appendix below). Users have the option to either use the default parameters, which include False Discovery Rate (FDR) for multiple comparisons adjustment, 0.5 as the absolute $\log_2$ Fold Change threshold, and 0.05 as the adjusted p-value threshold, or customize these values as needed. After selecting the desired parameters, the user must press the "Run the analysis" button and wait a few minutes for the analysis to be completed.

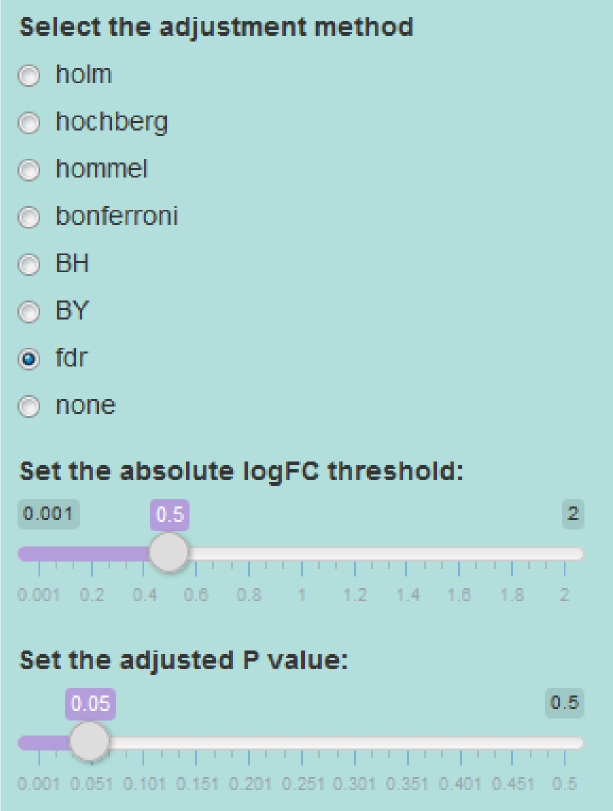


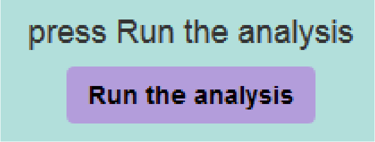


### b. Using DExplore with your own .CEL files

If the user wishes to use data that have not been submitted to the NCBI GEO, they can utilize the right side of the tab panel to browse their computer and select the files for analysis. The user should ensure that raw data is stored in .CEL format, with one file per experimental condition and replicate. After selecting the files and pressing the "Submit" button, DExplore provides a table showing information about the experimental design in the "Data Description" tab.

**Important notice:** When using uploaded data for analysis, users are encouraged to upload treated samples before control ones. This can be achieved by renaming the .CEL files accordingly, ensuring that treated samples precede control samples in the file list. Maintaining this order is crucial for accurately identifying DEGs, as DExplore analyzes data based on the order of samples provided. By prioritizing treated samples, DExplore can precisely identify upregulated and downregulated genes in accordance with the experimental design and treatment conditions.

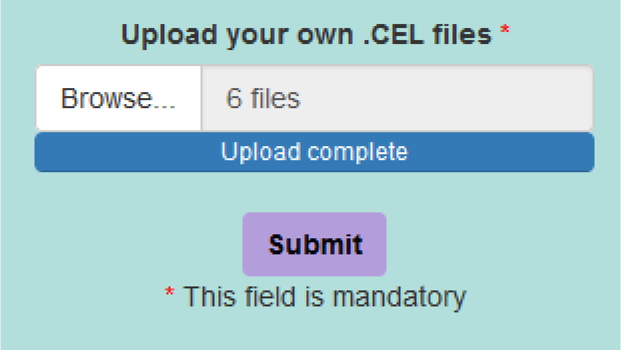


The user is required to fill in the table providing information about the experimental design, including specifying which samples consist the control data and identifying the treatment for treated samples. For treated samples, users must specify the type of treatment, duration, and concentration for chemical substances or dose for radiation, if applicable.

However, if duration and concentration do not affect the experimental design, users may leave these columns blank. Regardless, the columns for "treatment" and "replicate" must be filled out. After completing the table, users should press the "Save changes" button.

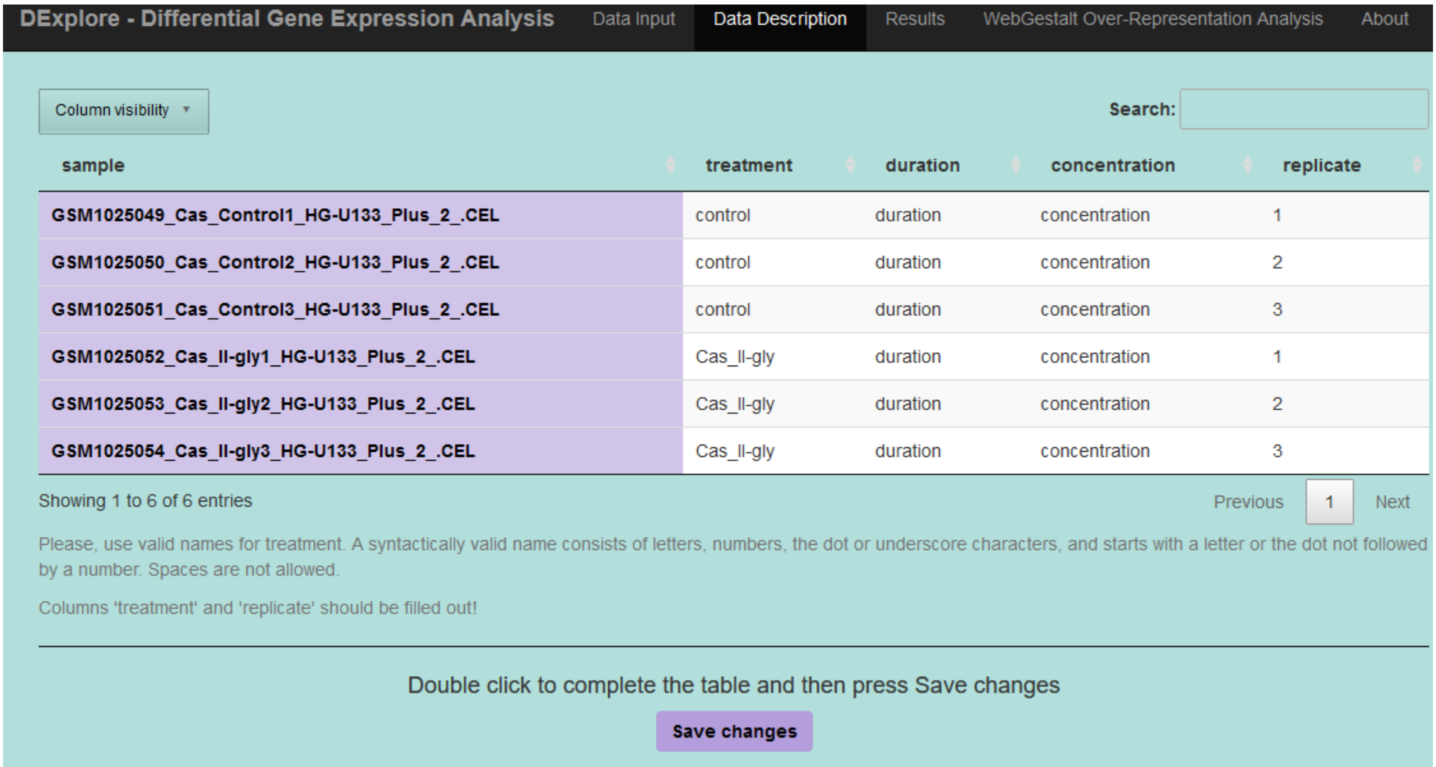


After saving the changes, the user must choose which of the possible comparisons will be carried out. For example, in cases involving two different treatment methods, A and B, along with control samples, the user may choose to (1) compare A to control, (2) compare B to control, or (3) compare A to B.

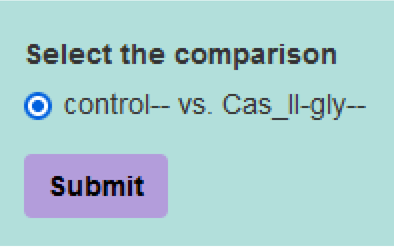


In the next step, users must select certain statistical parameters required for the analysis, including the method for adjusting the p-value for multiple comparisons, the absolute $\log_2$ Fold Change threshold, and the adjusted p-value threshold (see Appendix below). Users have the option to either use the default parameters, which include False Discovery Rate (FDR) for multiple comparisons adjustment, 0.5 as the absolute $\log_2$ Fold Change threshold, and 0.05 as the adjusted p-value threshold, or customize these values as needed. Once the parameters are set, users must press the 'Run the analysis' button and wait a few minutes for the analysis to be completed.

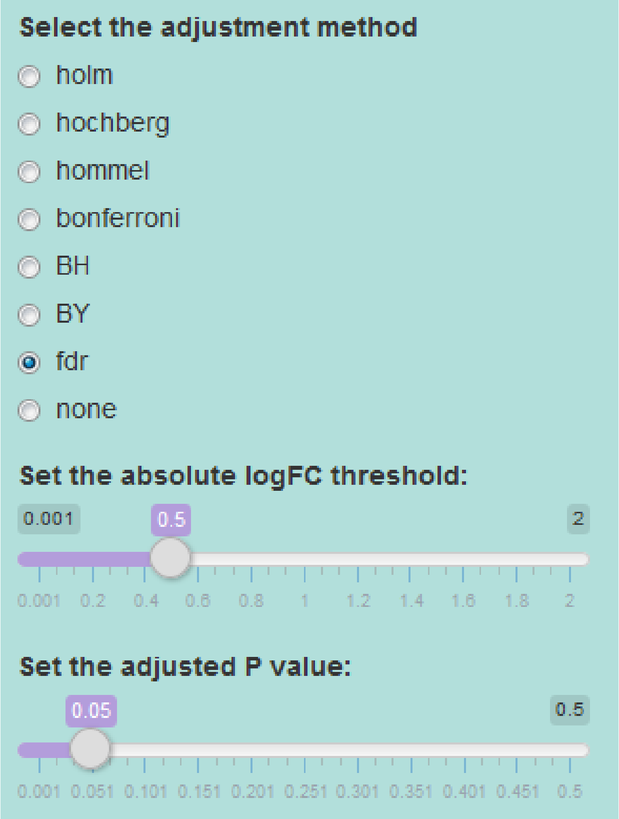
Select the adjustment method
holm
hochberg
hommel
bonferroni
BH
BY
fdr
none
Set the absolute logFC threshold:
0.001
0.5
2
0.001 0.2 0.4 0.6 0.8 1 1.2 1.4 1.6 1.8 2
Set the adjusted P value:
0.05
0.5
0.001 0.051 0.101 0.151 0.201 0.251 0.301 0.351 0.401 0.451 0.5

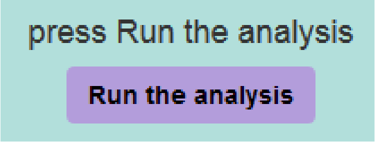
press Run the analysis
Run the analysis

## 3. Results

When the analysis is completed, in the "Results" tab the user can view a list of the differentially expressed genes (both the probe ID used by Affymetrix® and the gene symbol are provided) and some statistical values for each of them. In the gene symbol column, there is also a hyperlink to NCBI's Gene database, in case the user wishes to explore the genes on the list.

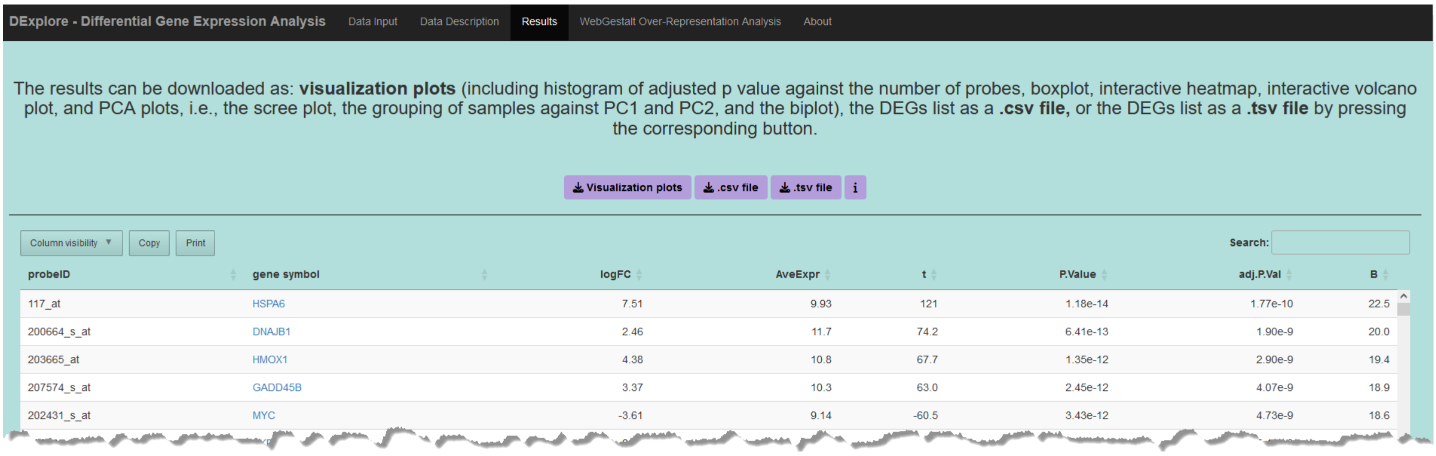


There is also the option to print the list (in order to save as a .pdf file), copy it to clipboard and then paste it in another file, or download the list as a .csv or a .tsv file in order to use it for further analyses.

Furthermore, users can easily download a zip file containing graphical representations of the Differential Expression Analysis by clicking the corresponding button.

## 4. WebGestalt Over-Representation Analysis

After detecting differentially expressed genes under two experimental conditions, it is common practice to proceed to functional enrichment analysis. DExplore facilitates this process by enabling users to perform functional enrichment analysis using the well-established web tool WebGestalt (www.webgestalt.org). By clicking the 'WebGestalt ORA' button, users are prompted to select one of the supported organisms from the WebGestalt platform and one of the reference sets to which the list of differentially expressed genes will be compared.

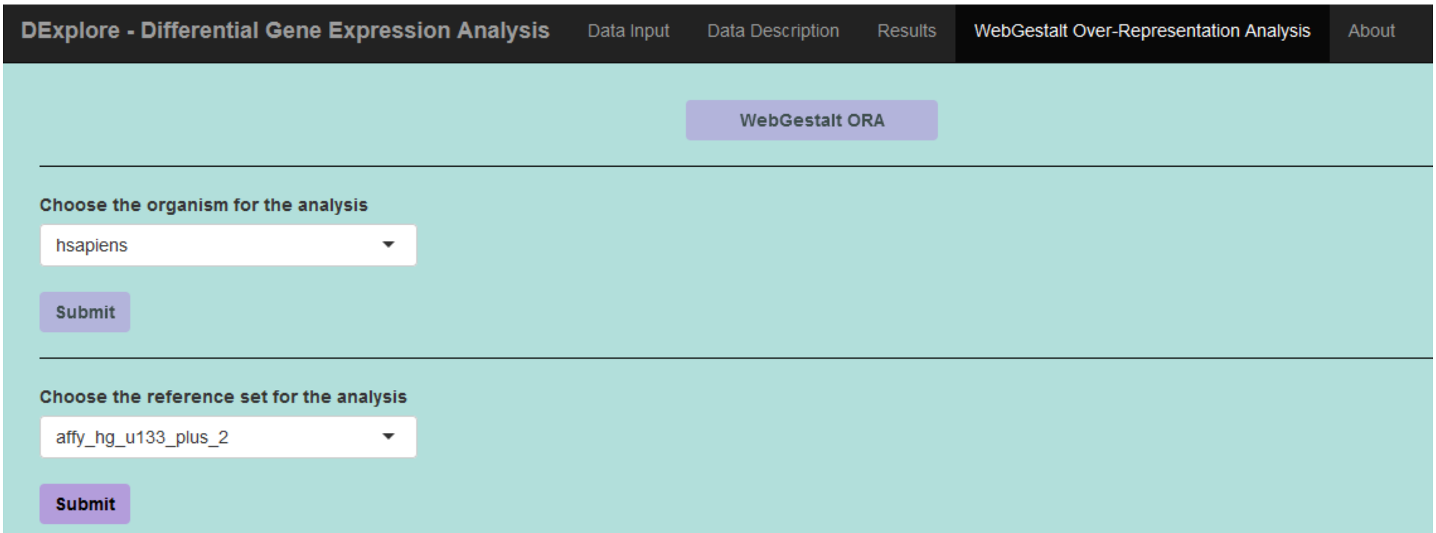


After a few minutes, DExplore renders the results of Over-Representation Analysis, which can be explored through a browser or downloaded to the user's computer for future use.

For more information regarding the WebGestalt web tool as well as the methods used for over-representation functional enrichment analysis, please visit www.webgestalt.org.

## 5. Appendix

### p-value and Multiple Comparisons

A p-value provides information about whether a statistical hypothesis test is significant or not and it also provides some indication on “how significant" the result is: the smaller the p-value the stronger the evidence against the null hypothesis. Most importantly, it does this without committing to a particular level of significance as traditional hypothesis tests and confidence intervals do[2].

In statistics, the multiple comparisons, multiplicity or multiple testing problem occurs when one considers a set of statistical inferences simultaneously or infers a subset of parameters selected based on the values observed. The more inferences are made the more likely erroneous inferences are to occur. Several statistical techniques have been developed to prevent this from happening, allowing significance levels for single and multiple comparisons to be directly compared. These techniques generally require a stricter significance threshold for individual comparisons, so as to compensate for the number of inferences being made.

### Adjustment for Multiple Comparisons

A typical microarray study generates a gene expression matrix with tens of thousands of rows—probe sets representing genes. Assuming we are performing 10,000 univariate tests on 10,000 genes with a significance level (α) of 0.05, we allow a 5% chance of making a Type I error, also known as a 'false positive' finding. This means that we expect 5% of the 10,000 genes, or 500 genes, to be deemed significant (differentially expressed) by chance alone. To control the overall probability of a Type I error, we must apply a correction for multiple testing. Whether we are repeatedly performing a t test, an ANOVA F test, or any other (univariate or multivariate) test resulting in a p-value, we must adjust the individual raw p-values for multiplicity in order to control the overall posterior false positive rate.

When performing multiple tests, rather than considering the significance level of individual tests, we should use a procedure that controls one of the Type I error rates defined for testing multiple null hypotheses. Among the commonly used Type I error rates are the family-wise error rate (FWER) and the false discovery rate (FDR).

Family-wise error rate (FWER)

The family-wise error rate is defined as the probability of at least one Type I error (i.e., at least one false positive) over all tests. This probability for a single test is equal to the significance level α of the test. However, if we perform M independent tests, this probability is equal to $1 - (1- \alpha)^M$, which for a high M is close to 1.

False discovery rate (FDR)

The false discovery rate is the expected proportion of false positives among the rejected null hypotheses (i.e., among all genes reported as differentially expressed). When all null hypotheses are true (i.e., none of the tested genes is differentially expressed), FDR is equal to FWER, but otherwise it is smaller.

Generally, procedures controlling the FWER are more conservative than those controlling FDR[3]. The best known among the FWER-controlling procedures are the classical single-step Bonferroni adjustment, the single-step Sidak procedure, and the step-down Holm procedure. The most popular among the FDR-controlling procedures is the step-up Benjamini and Hochberg procedure. The single-step procedures apply the same multiplicity adjustment to each individual α or raw p-value, whereas adjustments made by the stepwise approaches depend on the rank of the gene among all tested genes and on the outcomes of the tests for other genes.[4]

### Adjustment Methods provided by DExplore

DExplore allows you to select an adjustment method for your analysis among the Bonferroni correction ("bonferroni"), the correction introduced by Holm (1979)[5] ("holm"), by Hochberg (1988)[6] ("hochberg"), by Hommel (1988)[7] ("hommel"), by Benjamini & Hochberg (1995)[8] ("BH" or its alias "fdr"), and by Benjamini & Yekutieli (2001)[9] ("BY"). A pass-through option ("none") is also included.

The first four methods are designed to provide strong control of the family-wise error rate. There seems to be no reason to use the unmodified Bonferroni correction, since it is overridden by Holm's method, which is also valid under arbitrary assumptions.

Hochberg's and Hommel's methods are valid when the hypothesis tests are independent or when they are non-negatively associated.[10,11] Hommel's method is more powerful than Hochberg's, but the difference is usually small and the Hochberg p-values are faster to compute.

The "BH" (aka "fdr") and "BY" method of Benjamini, Hochberg, and Yekutieli control the false discovery rate, the expected proportion of false discoveries amongst the rejected hypotheses. The false discovery rate is a less stringent condition than the family-wise error rate, and thus these methods are more powerful than the rest.

For a more detailed review of adjustment methods commonly used, see Shaffer, J. P. Multiple Hypothesis Testing. *Annu. Rev. Psychol.* (1995). doi:10.1146/annurev.ps.46.020195.003021.

## 6. References

1. Liao, Y., Wang, J., Jaehnig, E. J., Shi, Z. & Zhang, B. WebGestalt 2019 : gene set analysis toolkit with revamped UIs and APIs. *Nucleic Acids Res.* **47**, 199–205 (2019).
2. Wright, S. P. Adjusted P-Values for Simultaneous Inference. *Biometrics* (1992). doi:10.2307/2532694
3. Dudoit, S. & Laan, M. J. van der. *Multiple Testing Procedures with Applications to Genomics*. *Springer* (2009). doi:10.1007/978-0-387-98135-2
4. Dziuda, D. M. *Data Mining for Genomics and Proteomics*. *Analysis of Gene and Protein Expression Data* (John Wiley & Sons, Inc., 2010). doi:10.1002/9780470593417
5. Holm, S. A Simple Sequentially Rejective Multiple Test Procedure. *Scand J Stat.* (1979). doi:10.2307/4615733
6. Hochberg, Y. A sharper bonferroni procedure for multiple tests of significance. *Biometrika* (1988). doi:10.1093/biomet/75.4.800
7. Hommel, G. A stagewise rejective multiple test procedure based on a modified bonferroni test. *Biometrika* (1988). doi:10.1093/biomet/75.2.383
8. Benjamini, Y. & Hochberg, Y. Controlling the false discovery rate: a practical and powerful approach to multiple testing. *J. R. Stat. Soc. Ser. B …* (1995). doi:10.2307/2346101
9. Benjamini, Y. & Yekutieli, D. The control of the false dicovery rate in multiple testing under depency. *Ann. Stat.* (2001).
10. Sarkar, S. K. Some probability inequalities for ordered MTP2 random variables: A proof of the Simes conjecture. *Ann. Stat.* (1998). doi:10.1214/aos/1028144846
11. Sarkar, S. K., Chang, C. K. & Chang, C. K. The simes method for multiple hypothesis testing with positively dependent test statistics. *J. Am. Stat. Assoc.* (1997). doi:10.1080/01621459.1997.10473682
