# Supplementary material for: DExplore: An Online Tool for Detecting Differentially Expressed Genes from mRNA Microarray Experiments
Source: Biology (Basel). 2024 May 16;13(5):351. doi: 10.3390/biology13050351 (PMC11117493; doi:10.3390/biology13050351)
Supplement: Supplementary file 1 [file biology-13-00351-s001.zip › S2. Tutorial for DExplore's Docker version.pdf]

Instructions for using DExplore's Docker version

To use DExplore from its Docker version, follow these steps:

1. Download and install Docker Desktop on your personal computer. Docker Desktop is a free application provided by Docker® and is compatible with Windows, Mac, and Linux operating systems. You can download it from https://www.docker.com/products/docker-desktop/.
2. Create a free account on Docker® to access Docker Hub. Docker Hub enables you to pull existing Docker images, such as DExplore, or push your own images.
3. After installing Docker Desktop and creating a Docker® account, you can pull the DExplore Docker image via Docker Desktop.

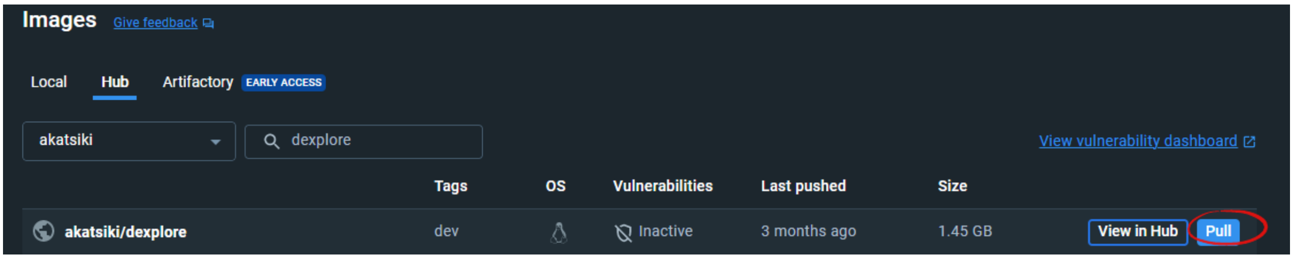

4. Running DExplore's Docker image is straightforward since Docker Desktop operates through a graphical user interface (GUI), eliminating the need for command-line inputs.

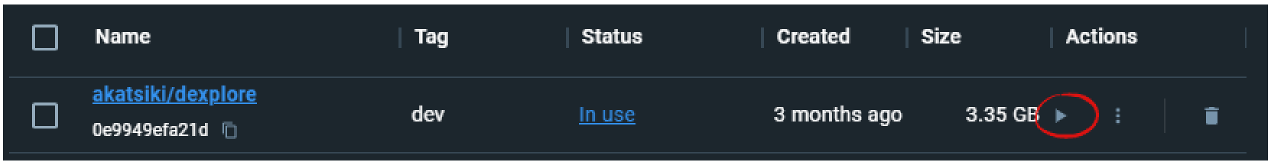

5. We strongly recommend users to configure optional settings as depicted in the following screenshot.

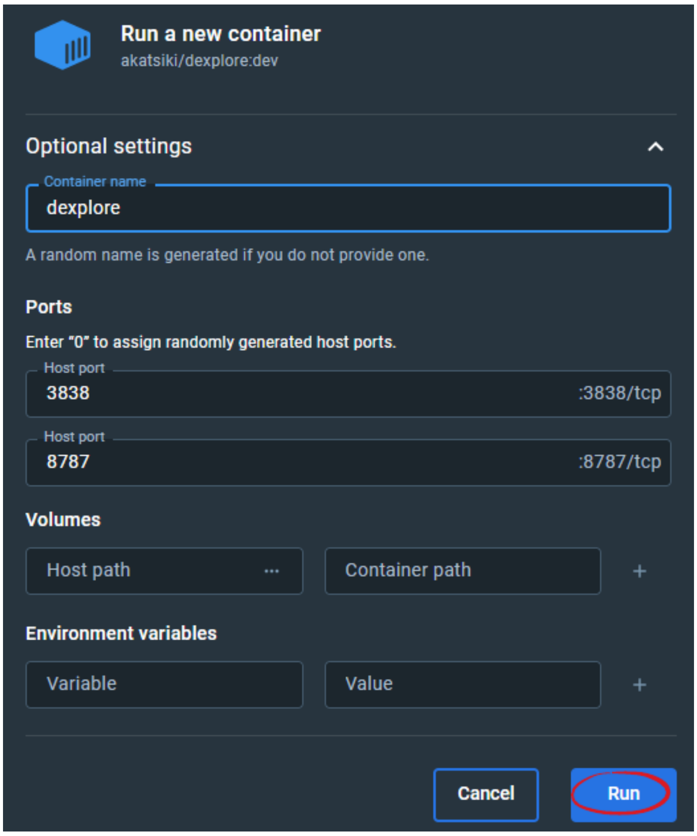

6. After configuring the settings, click the "Run" button. Open a new browser tab and enter "localhost:3838/dexplore/" in the address bar.
7. DExplore will run in the browser, mimicking its online version without any size restrictions or stability issues.

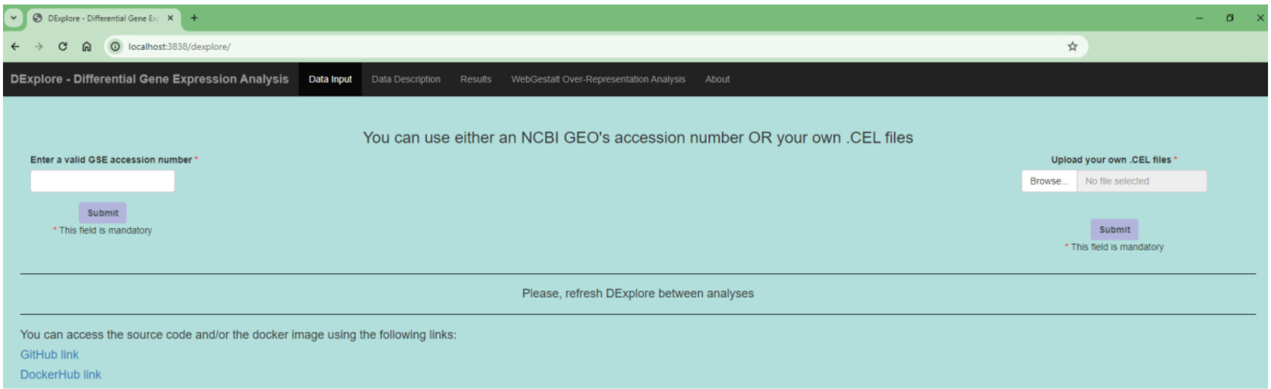

These steps ensure a smooth and user-friendly experience with DExplore's Docker version.

For troubleshooting, do not hesitate to contact us at dexplore.app[at]gmail.com.
