# Supplementary material for: DExplore: An Online Tool for Detecting Differentially Expressed Genes from mRNA Microarray Experiments
Source: Biology (Basel). 2024 May 16;13(5):351. doi: 10.3390/biology13050351 (PMC11117493; doi:10.3390/biology13050351)
Supplement: Supplementary file 1 [file biology-13-00351-s001.zip › S3. CARMAweb analysis.pdf]

Differential Expression Analysis: DExplore vs. CARMAweb

CARMAweb (Comprehensive R based Microarray Analysis web service) [1] is a web application designed for normalizing and analyzing data from microarray experiments conducted on major microarray platforms. The backend of CARMAweb utilizes the R programming language and Bioconductor packages, while the frontend is implemented in Java. As outlined in the CARMAweb user's guide, the analysis process involves uploading data, followed by preprocessing. During preprocessing, users can select the algorithm for preprocessing and subsequently proceed to detect differentially expressed genes. To access the analysis results, it is recommended to register for a free account. Upon registration, the results are stored for a few days, allowing users to conveniently access them by logging in. In contrast, users who choose not to register must wait for their analyses to conclude before gaining access to, exploring, or downloading their results.

For our comparisons, we utilized the most recent version of CARMAweb (release 1.6: March 2016), which is based on R version 3.2.3 with Bioconductor 3.2 (https://carmaweb.genome.tugraz.at/carma/, accessed on 2 March 2024).

For the comparisons, we utilized the same datasets discussed in the main text, namely GSE39870 [2] and GSE113427 [3,4], which were also employed for the comparison of DExplore and GEO2R. The preprocessing algorithm chosen was rma (Robust Multiarray Analysis) [5,6], and for the detection of differentially expressed genes, we used moderated t-statistics (limma) [7]. To correct for multiple testing, we employed the Benjamini & Hochberg step-up FDR controlling procedure [8], consistent with the approach in DExplore.

Results for the comparison using the dataset with accession number GSE39870

As reported in the main text, DExplore identified 739 differentially expressed genes, whereas CARMAweb detected 1055 differentially expressed genes. Notably, 681 genes were identified as differentially expressed by both DExplore and CARMAweb, with 410 showing downregulation and 271 displaying upregulation (Figure S1). The complete list of the identified DEGs from both analyses are available in supplementary material (Tables S1, S3 and S7-S9).

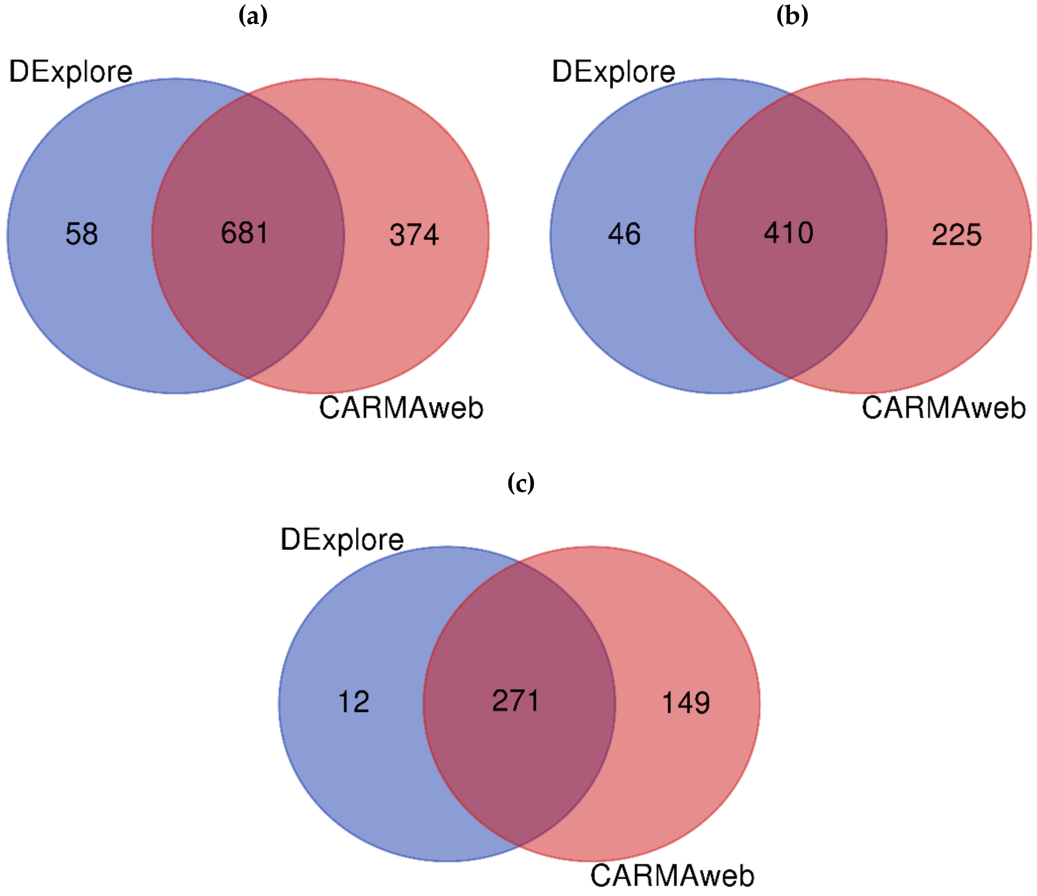

Figure S1. Venn diagrams showing (a) all the differentially expressed genes, (b) the under-expressed genes, and (c) the over-expressed genes following treatment with doxorubicin identified by DExplore (light navy blue) and CARMAweb (coral pink). To

draw the diagrams, we used the "Venn Diagrams" online tool available at https://bioinformatics.psb.ugent.be/webtools/Venn/, accessed on 17 April 2024.

As in the DExplore vs. GEO2R comparison, we conducted Functional Enrichment Analysis using WebGestalt's Over-Representation Analysis (ORA) [9]. The parameters employed for ORA were the same as in the main text (Figure 6).

WebGestalt's ORA identified 359 enriched GO terms using all DEGs detected with DExplore. The same input and statistical parameters were used for the analysis with CARMAweb and resulted in 361 enriched GO terms. As depicted in Figure S2, both analyses shared 322 common enriched GO terms. Detailed results, along with comparisons between DExplore and CARMAweb, considering separately under- or over-expressed DEGs, are available in the appendix (Tables S10-S24).

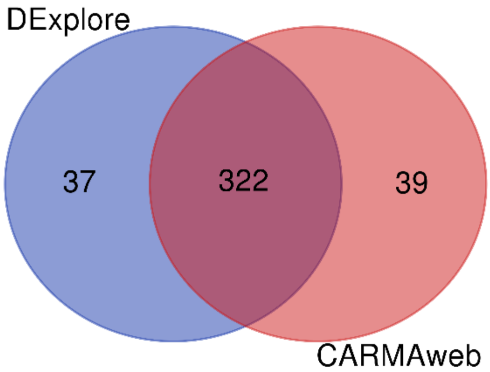

Figure S2. Venn diagram showing the comparison of enriched Gene Ontology terms derived by WebGestalt Over-Representation Analysis (ORA) using all differentially expressed genes detected by DExplore (light navy blue) and CARMAweb (coral pink). To draw the diagrams, we used the "Venn Diagrams" online tool available at https://bioinformatics.psb.ugent.be/webtools/Venn/, accessed on 17 April 2024.

Results for the comparison using the dataset with accession number GSE113427

The analysis conducted on the GSE113427 [3,4] dataset mirrored the procedure detailed earlier. In this case, DExplore identified 237 differentially expressed genes, while CARMAweb pinpointed 181 genes with differential expression. Comparison of the outcomes revealed that 114 genes were commonly identified as differentially expressed by both DExplore and CARMAweb, with 67 showing downregulation and 47 exhibiting upregulation (Figure S3). The complete list of the identified DEGs from both analyses are available in supplementary material (Tables S25, S27 and S31-33).

It is important to note that CARMAweb automatically annotated the probeset IDs indicating differential expression using Ensembl's gene identifiers without requiring user input for this annotation process. To facilitate comparison, we employed g:Convert, a tool provided by g:Profiler [10], to convert Ensembl gene identifiers to gene symbols. During this process, 18 Ensembl gene identifiers could not be mapped to gene symbols and were consequently excluded from the analysis.

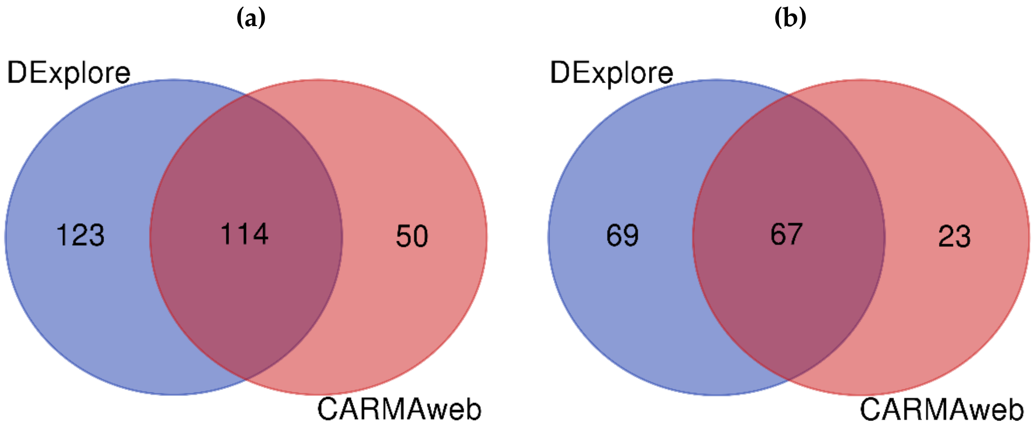

(c)

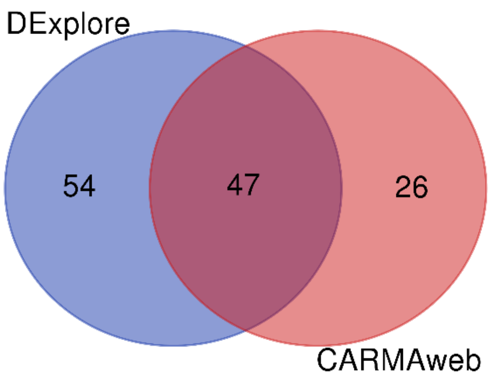

Figure S3. Venn diagrams showing (a) all the differentially expressed genes, (b) the under-expressed genes, and (c) the over-expressed genes following treatment with doxorubicin identified by DExplore (light navy blue) and CARMAweb (coral pink). To draw the diagrams, we used the "Venn Diagrams" online tool available at [https://bioinformatics.psb.ugent.be/webtools/Venn/, accessed on 17 April 2024](https://bioinformatics.psb.ugent.be/webtools/Venn/).

For the Functional Enrichment Analysis using WebGestalt's Over-Representation Analysis (ORA), the parameters employed for ORA are shown in Figure S4.

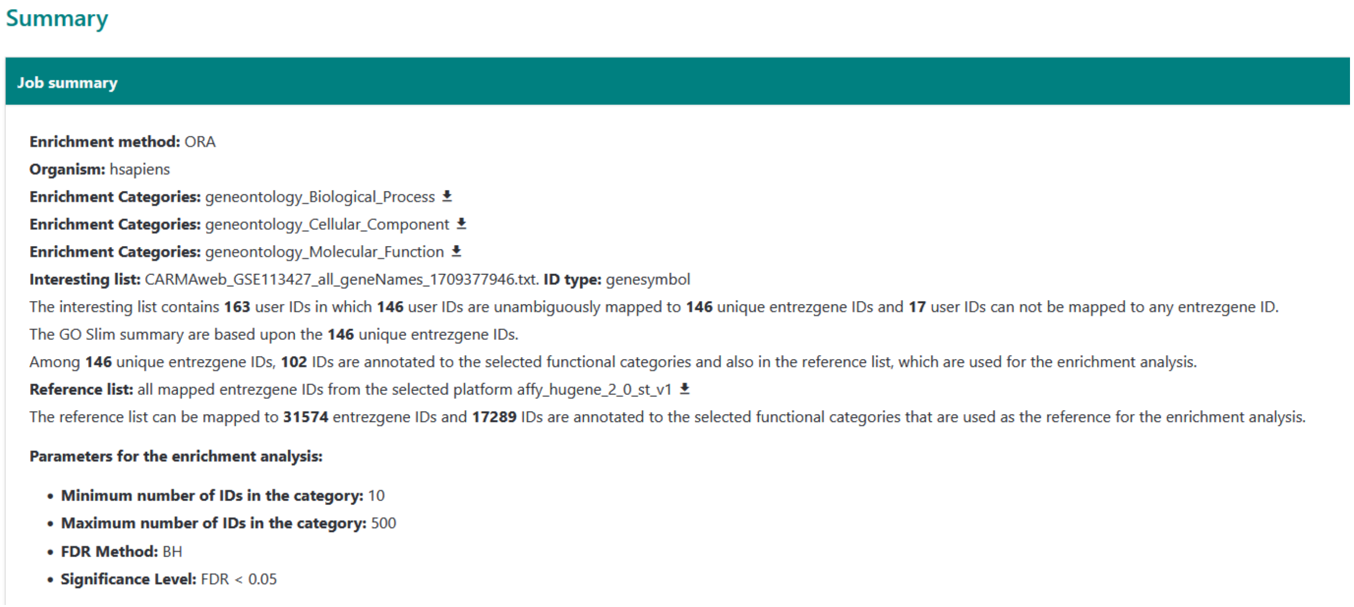
Summary

Job summary

Enrichment method: ORA
Organism: hsapiens
Enrichment Categories: geneontology_Biological_Process
Enrichment Categories: geneontology_Cellular_Component
Enrichment Categories: geneontology_Molecular_Function
Interesting list: CARMAweb_GSE113427_all_geneNames_1709377946.txt. **ID type:** genesymbol
The interesting list contains **163** user IDs in which **146** user IDs are unambiguously mapped to **146** unique entrezgene IDs and **17** user IDs can not be mapped to any entrezgene ID.
The GO Slim summary are based upon the **146** unique entrezgene IDs.
Among **146** unique entrezgene IDs, **102** IDs are annotated to the selected functional categories and also in the reference list, which are used for the enrichment analysis.
Reference list: all mapped entrezgene IDs from the selected platform affy_hugene_2_0_st_v1
The reference list can be mapped to **31574** entrezgene IDs and **17289** IDs are annotated to the selected functional categories that are used as the reference for the enrichment analysis.

Parameters for the enrichment analysis:

- **Minimum number of IDs in the category:** 10
- **Maximum number of IDs in the category:** 500
- **FDR Method:** BH
- **Significance Level:** FDR < 0.05

Figure S4. The parameters used for WebGestalt Over-Representation Analysis (ORA) of GSE113427.

Due to CARMAweb's automated annotation, we utilized gene symbols as input for the Over-Representation Analysis for both DExplore's and CARMAweb's lists of differentially expressed genes so as the results of the analysis to be comparable. This led to slight variations in the results of the ORA analysis using DExplore's DEGs, as compared to those from DExplore versus GEO2R comparison described in the main text.

WebGestalt's ORA identified 101 enriched GO terms using all DEGs detected with DExplore. The same input and statistical parameters were used for the analysis with CARMAweb and resulted in 80 enriched GO terms. As depicted in Figure S5, both analyses shared 69 common enriched GO terms. Detailed results, along with comparisons between DExplore and CARMAweb, considering separately under- or over-expressed DEGs, are available in the appendix (Tables S34-S47).

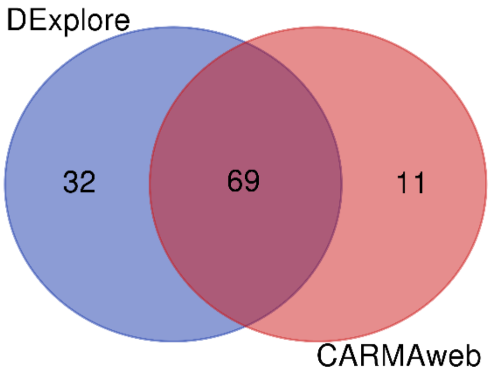

Figure S5. Venn diagram showing the comparison of enriched Gene Ontology terms derived by WebGestalt Over-Representation Analysis (ORA) using all differentially expressed genes detected by DExplore (light navy blue) and CARMAweb (coral pink). To draw the diagrams, we used the "Venn Diagrams" online tool available at https://bioinformatics.psb.ugent.be/webtools/Venn/, accessed on 17 April 2024.

It is worth noting that when utilizing the 73 upregulated genes identified by CARMAweb as input for ORA, no significant results were obtained under the threshold FDR < 0.05. In contrast, using DExplore's and GEO2R's inputs resulted in 4 and 2 enriched GO terms, respectively.

References

1. Rainer, J.; Sanchez-Cabo, F.; Stocker, G.; Sturn, A.; Trajanoski, Z. CARMAweb : Comprehensive R- and Bioconductor-Based Web Service for Microarray Data Analysis. *Nucleic Acids Res.* **2006**, *34*, 498–503, doi:10.1093/nar/gkl038.
2. Bailey, S.T.; Shin, H.; Westerling, T.; Liu, X.S.; Brown, M. Estrogen Receptor Prevents P53-Dependent Apoptosis in Breast Cancer. *Proc. Natl. Acad. Sci. U. S. A.* **2012**, *109*, 18060–18065, doi:10.1073/pnas.1018858109.
3. Newell, M.; Brun, M.; Field, C.J. Treatment with DHA Modifies the Response of MDA-MB-231 Breast Cancer Cells and Tumors from Nu/Nu Mice to Doxorubicin through Apoptosis and Cell Cycle Arrest. *J. Nutr.* **2019**, *149*, 46–56, doi:10.1093/jn/nxy224.
4. Ewaschuk, J.B.; Newell, M.; Field, C.J. Docosahexanoic Acid Improves Chemotherapy Efficacy by Inducing CD95 Translocation to Lipid Rafts in ER2 Breast Cancer Cells. *Lipids* **2012**, *47*, 1019–1030, doi:10.1007/s11745-012-3717-7.
5. Irizarry, R.A.; Hobbs, B.; Collin, F.; Beazer-Barclay, Y.D.; Antonellis, K.J.; Scherf, U.; Speed, T.P.; Beazer-Barclay, Y.D.; Antonellis, K.J.; Scherf, U.; et al. Exploration, Normalization, and Summaries of High Density Oligonucleotide Array Probe Level Data. *Biostatistics* **2003**, *4*, 249–264, doi:10.1093/biostatistics/4.2.249.
6. Irizarry, R.A.; Ooi, S.L.; Wu, Z.; Boeke, J.D. Use of Mixture Models in a Microarray-Based Screening Procedure for Detecting Differentially Represented Yeast Mutants. *Stat. Appl. Genet. Mol. Biol.* **2003**, *2*, doi:10.2202/1544-6115.1002.
7. Ritchie, M.E.; Phipson, B.; Wu, D.; Hu, Y.; Law, C.W.; Shi, W.; Smyth, G.K. Limma Powers Differential Expression Analyses for RNA-Sequencing and Microarray Studies. *Nucleic Acids Res.* **2015**, *43*, e47, doi:10.1093/nar/gkv007.
8. Benjamini, Y.; Hochberg, Y. Controlling the False Discovery Rate: A Practical and Powerful Approach to Multiple Testing. *J. R. Stat. Soc. Ser. B …* **1995**, doi:10.2307/2346101.
9. Liao, Y.; Wang, J.; Jaehnig, E.J.; Shi, Z.; Zhang, B. WebGestalt 2019: Gene Set Analysis Toolkit with Revamped UIs and APIs. *Nucleic Acids Res.* **2019**, *47*, W199–W205, doi:10.1093/nar/gkz401.
10. Kolberg, L.; Raudvere, U.; Kuzmin, I.; Adler, P.; Vilo, J.; Peterson, H. G:Profiler—Interoperable Web Service for Functional Enrichment Analysis and Gene Identifier Mapping (2023 Update). *Nucleic Acids Res.* **2023**, *51*, W207–W212, doi:10.1093/nar/gkad347.
